# Strong conductivity enhancement of La-doped BaSnO_3_ transparent films on Al_2_O_3_ with the assistance of templated epitaxy for electromagnetic shielding in extreme environments

**DOI:** 10.1186/s40580-023-00355-9

**Published:** 2023-02-15

**Authors:** Youngkyoung Ha, Shinbuhm Lee

**Affiliations:** Department of Physics and Chemistry, Department of Emerging Materials Science, DGIST, Daegu, 42988 Republic of Korea

**Keywords:** Transparent conductors, La-doped BaSnO_3_, Templated epitaxy, Al_2_O_3_, Single-crystalline films

## Abstract

Transparent La-doped BaSnO_3_ (BLSO) epitaxial films grown on expensive perovskites show promising conductive properties. However, BLSO films on Al_2_O_3_ have a higher sheet resistance by two orders of magnitude. In this research, the sheet resistance of BLSO films is recovered to that of the single-crystalline level by growing (111)-oriented BLSO epitaxial films on (0001)Al_2_O_3_ with the assistance of (111)BaZrO_3_/MgO template bilayer. Their intriguing transparent conductive properties, including high electromagnetic shielding effectiveness (~ 13.2 dB at 10 GHz) and high stability at 700 °C, will promote stable optoelectronic applications in extreme environments with economic benefits.

**Graphical Abstract:**

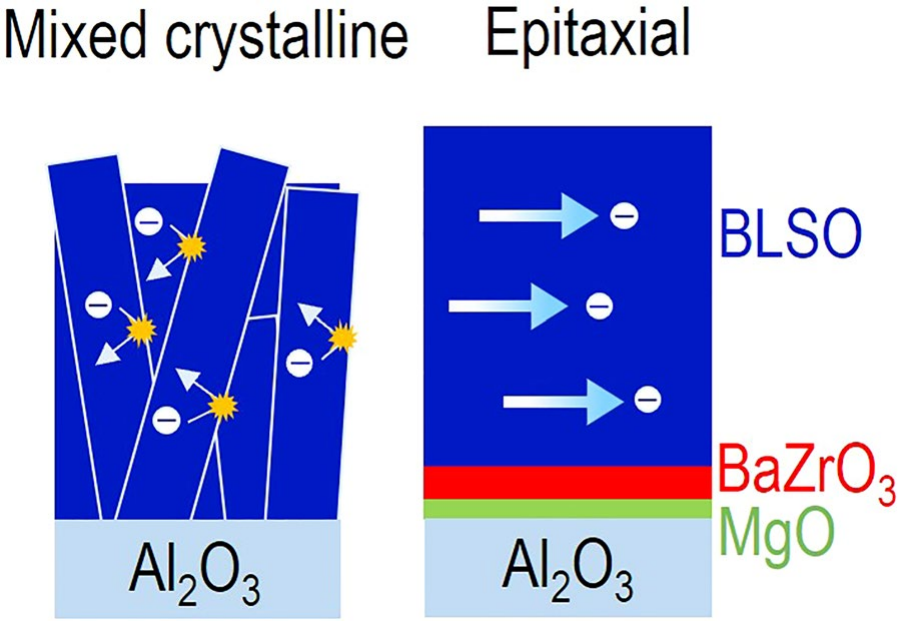

**Supplementary Information:**

The online version contains supplementary material available at 10.1186/s40580-023-00355-9.

## Introduction

Transparent conductors (TCs) are key components of modern optoelectronics [1–7]. The discovery of La-doped BaSnO_3_ (BLSO) has rejuvenated interest in TCs [8–11]. BaSnO_3_ is a wide-bandgap oxide, whose bandgap of 3.3–4.1 eV is determined by charge transfer from the valence band of the O 2*p* orbitals to the conduction band of the Sn 5*s* orbital [8, 9]. Aliovalent cation (e.g., La^3+^) doping renders Ba^2+^Sn^4+^O_3_ conductive, analogous to the prototypical wide-bandgap TC Sn^4+^-doped ${\mathrm{In}}_{2}^{3+}{\mathrm{O}}_{3}$. The straight O–Sn–O connectivity and large Sn 5*s* orbital in the cubic perovskite structure provide a dispersive conduction band with a small effective mass, resulting in a high electron mobility of ~ 250 cm^2^ V^−1^ s^−1^ in single crystals [8, 9] and ~ 100 cm^2^ V^−1^ s^−1^ in films [9, 10] at room temperature. The thermal stability of BLSO might enable optoelectronic applications in extreme environments [12]. Methylammonium lead iodide-based solar cells fabricated with colloidally prepared BLSO electrodes have achieved a high power conversion efficiency of 21.2% [13]. Irrespective of such merits, BLSO has rarely been examined for the simultaneous attainment of low sheet resistance, high transmittance in the visible region, high electromagnetic shielding effectiveness, and high stability, which have been critical bottlenecks in optoelectronic technology.

The resistivity of BLSO is highly affected by crystallinity, where BLSO single crystals have a much lower resistivity by 2–3 orders of magnitude than polycrystalline specimens [8, 9]. Hence, the majority of studies concerning BLSO films have reported the properties of epitaxial films grown on cubic substrates, e.g., SrTiO_3_, KTaO_3_, and MgO [14–18]. The similar cubic structure between the BLSO film and substrates enables cube-on-cube epitaxial growth due to a moderate lattice mismatch ($=\frac{{a}_{\mathrm{substrate}}-{a}_{\mathrm{film}}}{{a}_{\mathrm{film}}}\times 100$) between −5.22 and 1.94% along the [100]BLSO || [100] substrate, where *a*_substrate_ denotes the lattice parameters of SrTiO_3_ (*a* = *b* = *c* = 3.905 Å), KTaO_3_ (3.99 Å), and MgO (4.20 Å) substrates and *a*_film_ represents the lattice parameter of BaSnO_3_ (4.12 Å) films (see Table 1 for a summary of the crystal structures and lattice parameters of the materials used in this work). However, SrTiO_3_ and KTaO_3_ are quite expensive, and hygroscopic MgO tends to absorb water molecules from the environment [19]. On the other hand, Al_2_O_3_ (*a* = *b* = 4.76 Å, *c* = 12.99 Å, *γ* = 120°) is low cost, available in large wafer form, chemically, thermally, and mechanically stable, and highly transparent over a wide spectrum. Consequently, it is used as a conventional substrate for optoelectronics. Therefore, it is highly desirable to characterize the electrical and optical properties of BLSO films grown on Al_2_O_3_.

**Table 1:** Materials, crystal structures, lattice parameters, and epitaxial relationships among the La-doped BaSnO_3_ (BLSO) film, template layers, and (0001)-oriented Al_2_O_3_ substrate, and in-plane lattice mismatches

	Materials	Crystal structures (space group)	Lattice parameters	Epitaxial relationships among BLSO film, template layers, and (0001)Al_2_O_3_ in-plane lattice mismatches (%)
Film	La-doped BaSnO_3_ (BLSO)	Cubic perovskite $(Pm\overline{3 }m)$	*a* = 4.12 Å for BaSnO_3_	–
Substrate	Al_2_O_3_	Hexagonal $(R\overline{3 }c)$	*a* = *b* = 4.76 Å, *c* = 12.99 Å *γ* = 120^o^	–
Template layer	BaZrO_3_/MgO	Cubic perovskite $(Pm\overline{3 }m)$ Cubic halite $(Fm\overline{3 }m)$	*a* = 4.19 Å *a* = 4.20 Å	(111)BLSO || (111)BaZrO_3_ || (111)MgO || (001)Al_2_O_3_ $[1\overline{1 }0]$ MgO || [100]Al_2_O_3_, lattice mismatch −19.9% (0.2% for 4:5 MgO:Al_2_O_3_) $[1\overline{1 }0]$ BaZrO_3_ || [$[1\overline{1 }0]$ MgO, 0.2% $[1\overline{1 }0]$ BLSO || $[1\overline{1 }0]$ BaZrO_3_, 1.7%
Template layer	MgO	Cubic halite $(Fm\overline{3 }m)$	*a* = 4.20 Å	(111)BLSO || (111)MgO || (001)Al_2_O_3_ $[1\overline{1 }0]$ MgO || [100]Al_2_O_3_, lattice mismatch −19.9% (0.2% for 4:5 MgO:Al_2_O_3_) $[1\overline{1 }0]$ BLSO || $[1\overline{1 }0]$ MgO, 1.9%
Template layer	Y-stabilized ZrO_2_ (YSZ)	Cubic fluorite $(Fm\overline{3 }m)$	*a* = 5.13 Å	(011)BLSO || (111)YSZ || (001)Al_2_O_3_ $[1\overline{1 }0]$ YSZ || [100]Al_2_O_3_, lattice mismatch −34.4% (−1.6% for 2:3 YSZ:Al_2_O_3_) [100]BLSO || $[1\overline{1 }0]$ YSZ, −11.9%
Template layer	Gd-doped CeO_2_ (GDC)	Cubic fluorite $(Fm\overline{3 }m)$	*a* = 5.41 Å	(011)BLSO || (111)GDC || (001)Al_2_O_3_ $[\overline{1 }\overline{1 }2]$ GDC || [100]Al_2_O_3_, 7.7% $[0\overline{1 }1]$ BLSO || $[\overline{1 }\overline{1 }2]$ GDC, −19.5%

Here, we found that the sheet resistance of BLSO films grown on Al_2_O_3_ was much higher by two orders of magnitude than that of single-crystalline films grown on perovskites. These poor properties might be attributed to the hexagonal structure of Al_2_O_3_ substrates hindering the single-crystalline growth of cubic perovskite BLSO epitaxial films. However, the sheet resistance was recovered to the single-crystalline level by epitaxially growing (111)-oriented BLSO films on (0001)Al_2_O_3_ with (111)BaZrO_3_/MgO template bilayer. Using our BLSO epitaxial films, we could measure the ultraviolet transmittance, electromagnetic shielding effectiveness, and thermal stability, which have rarely been investigated. To understand the origin of the enhanced crystallinity, we carried out X-ray diffraction (XRD) and transmission electron microscopy (TEM). Hereafter, we sometimes use the simpler form ${\mathrm{BLSO}}_{\mathrm{substrate}}^{\mathrm{template \;   layers}}$, e.g., ${\mathrm{BLSO}}_{(0001){\mathrm{Al}}_{2}{\mathrm{O}}_{3}}^{{\mathrm{BaZrO}}_{3}/\mathrm{MgO}}$, for the BLSO films on (0001)Al_2_O_3_ with BaZrO_3_/MgO template bilayer.

## Result and discussion

### Epitaxial stabilization of La-doped BaSnO_3_ (BLSO) films on (0001)-oriented Al_2_O_3_ with BaZrO_3_/MgO template bilayer

**Fig. 1:**
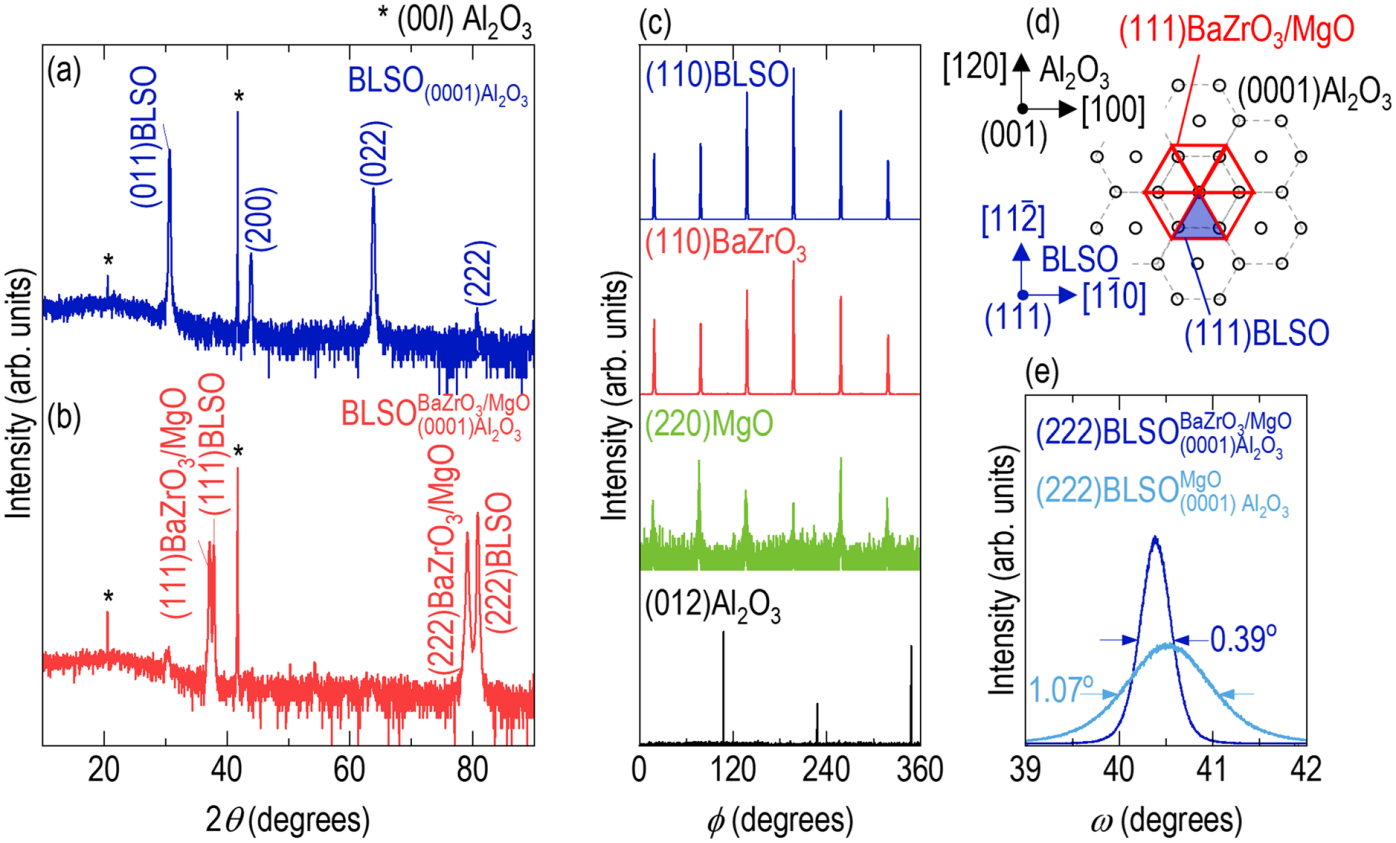
Epitaxial stabilization of BLSO films on (0001)Al_2_O_3_ with BaZrO_3_/MgO template bilayer. For convenience, the simpler form of ${\mathrm{BLSO}}_{\mathrm{substrate}}^{\mathrm{template \; layers}}$ is used. **a** The (110), (200), (220), and (222) diffraction peaks in the X-ray diffraction (XRD) *θ–2θ* scan of ${\mathrm{BLSO}}_{(0001){\mathrm{Al}}_{2}{\mathrm{O}}_{3}}$ indicate the formation of mixed-crystalline phases. The asterisks indicate the (003) and (006) diffraction peaks of Al_2_O_3_. **b** The XRD *θ–2θ* scan of ${\mathrm{BLSO}}_{(0001){\mathrm{Al}}_{2}{\mathrm{O}}_{3}}^{{\mathrm{BaZrO}}_{3}/\mathrm{MgO}}$ shows the (111) and (222) diffraction peaks of BaZrO_3_/MgO and BLSO, indicating the formation of (111)-oriented BLSO epitaxial films on (0001)Al_2_O_3_ with (111)BaZrO_3_/MgO template bilayer. **c** The XRD *ϕ* scans of (110)BLSO, (110)BaZrO_3_, and (220)MgO show six diffraction peaks separated by 30° from (012)Al_2_O_3_, corresponding to three-fold symmetric in-plane matching, as schematically shown in **d**. **e** The full-width at half-maximum in the XRD *ω*-scans decrease from 1.07° in ${\mathrm{BLSO}}_{(0001){\mathrm{Al}}_{2}{\mathrm{O}}_{3}}^{\mathrm{MgO}}$ to 0.39° in ${\mathrm{BLSO}}_{(0001){\mathrm{Al}}_{2}{\mathrm{O}}_{3}}^{{\mathrm{BaZrO}}_{3}/\mathrm{MgO}}$, indicating an improved crystallinity of the BLSO epitaxial films with the use of the BaZrO_3_ template layer

The direct growth of BLSO films on Al_2_O_3_ resulted in poor crystallinity. The XRD *θ* –2*θ* scans revealed a mixture of (110), (200), (220), and (222) diffraction peaks for BLSO films grown on (0001)-oriented Al_2_O_3_ (Fig. 1a). Scans of the BLSO films on $(1\overline{1 }02)$-, $(11\overline{2 }0)$-, and $(10\overline{1 }0)$-oriented Al_2_O_3_ showed weak diffraction peaks corresponding to mixed crystallographic orientations (Additional file 1: Fig. S1). This growth of non-epitaxial films was mainly the result of an incommensurate interface between cubic perovskite BLSO and hexagonal Al_2_O_3_. In addition, the difference between the thermal expansions of BLSO and Al_2_O_3_ could contribute to non-epitaxial growth, as observed between perovskite oxides and Al_2_O_3_. [20, 21]

Epitaxy with the assistance of template layers, so-called templated epitaxy, is a simple way to grow single-crystalline films when the film and substrate have an incommensurate interface. For example, a Y-stabilized ZrO_2_ (YSZ) template layer has been used to integrate epitaxial films (e.g., ferroelectric Bi_3.25_La_0.75_Ti_3_O_12_ and Y-doped HfO_2_) on silicon [22, 23]. TiO_2_(B) epitaxial films were successfully grown on (001)-oriented SrTiO_3_ using the VO_2_(B) template layer, while anatase TiO_2_ epitaxial films were formed without the template layer [24]. Figure 1b shows an XRD *θ* –2*θ* scan of BLSO films on (0001)Al_2_O_3_ with BaZrO_3_/MgO template bilayer. Different from the direct growth of BLSO films on (0001)Al_2_O_3_ (Fig. 1a), four strong peaks were observed at 37.2°, 37.8°, 79.1°, and 80.8°, which were due to diffraction from the (111) and (222) planes of BaZrO_3_/MgO and BLSO, respectively. (111)BLSO epitaxial films were successfully deposited on (0001)Al_2_O_3_ with the help of (111)BaZrO_3_/MgO epitaxial template bilayer.

XRD *ϕ* scans were carried out to establish the role of an MgO layer in the epitaxial transformation of BLSO films on (0001)Al_2_O_3_. These scans provided information on in-plane matching between (111)MgO and (0001)Al_2_O_3_. As shown at the bottom of Fig. 1c, hexagonal Al_2_O_3_ showed three diffraction peaks corresponding to the (012) plane with a uniform *ϕ* interval of 120°. The *ϕ* scan of (220)MgO exhibited six strong diffraction peaks separated by 30° from those of (012)Al_2_O_3_. This observation indicates the in-plane matching of $[1\overline{1 }0]$ MgO || [100]Al_2_O_3_, as schematically shown in Fig. 1d. Considering the lattice parameters of MgO and Al_2_O_3_, the lattice mismatch along $[1\overline{1 }0]$ MgO || [100]Al_2_O_3_ was −19.9%, and an alternative alignment of a 4:5 lattice ratio of MgO:Al_2_O_3_ significantly reduced the mismatch to 0.2% [25], which behaviour has been widely observed elsewhere [26]. The six strong diffraction peaks observed in the *ϕ* scans of (110)BLSO and (110)BaZrO_3_ at the same *ϕ* angles of (220)MgO indicated that the BLSO film and cubic BaZrO_3_ (lattice parameters of 4.19 Å) template layer were cube-on-cube aligned on the MgO template layer due to similar lattice parameters. Figure 1e shows the XRD *ω* scans of (222)BLSO with and without the BaZrO_3_ template layer. By inserting a BaZrO_3_ layer between the BLSO film and MgO layer, the full-width at half-maximum (FWHM) of peaks in the *ω* scans decreased from 1.07° to 0.39° since the surfaces of BLSO and BaZrO_3_ have a similar atomic arrangement, and the lattice mismatch decreased from 1.9% for BLSO/MgO to 1.7% for BLSO/BaZrO_3_. Therefore, while the MgO template layer played a key role in the epitaxial growth of BLSO films on (0001)Al_2_O_3_, the BaZrO_3_ template layer further enhanced the crystallinity of the BLSO films. Table 1 summarizes the epitaxial relationships among the BLSO film, template layers, and (0001)Al_2_O_3_ substrate and the in-plane lattice mismatches.

**Fig. 2:**
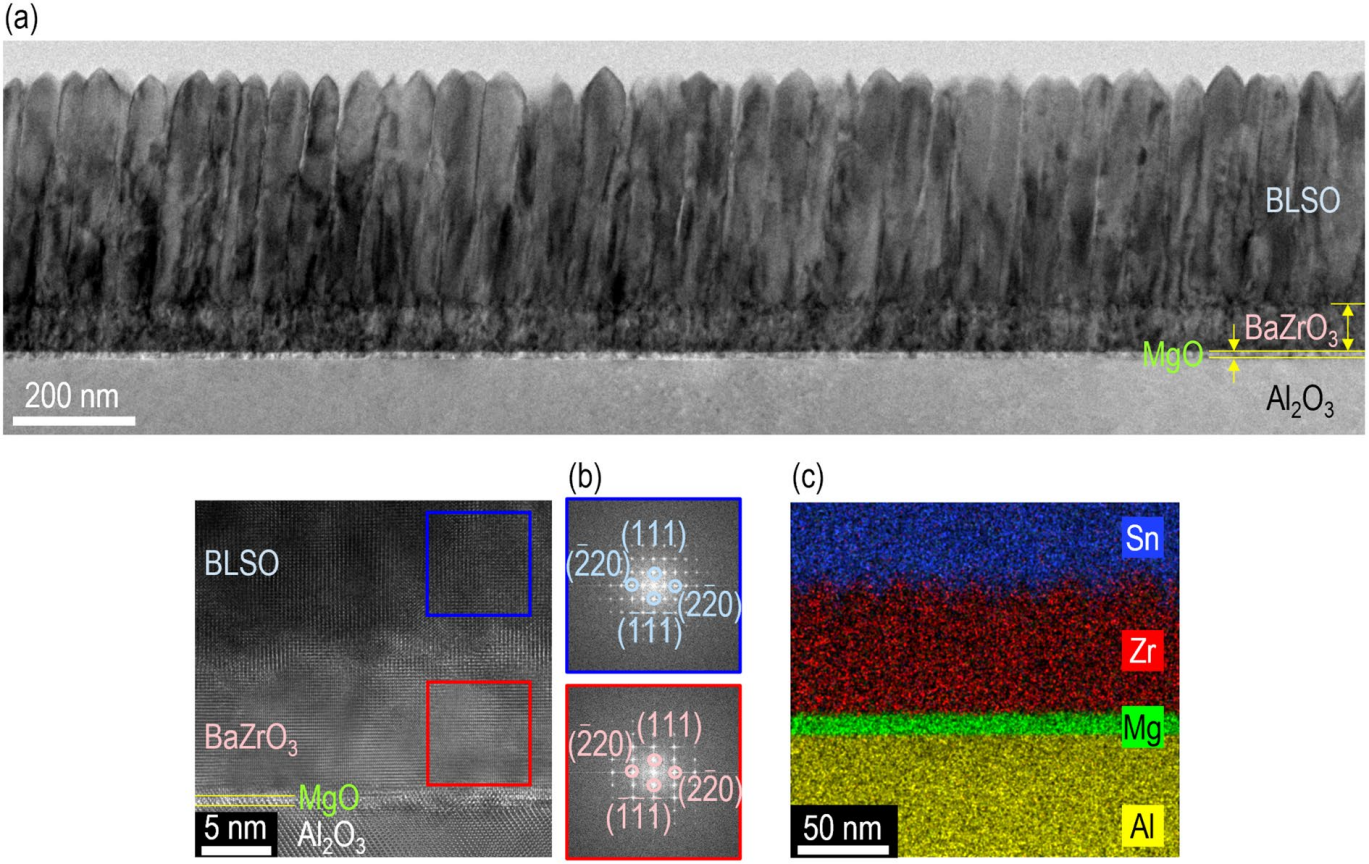
Nanoscopic investigation of the crystal structure of ${\mathrm{BLSO}}_{(0001){\mathrm{Al}}_{2}{\mathrm{O}}_{3}}^{{\mathrm{BaZrO}}_{3}/\mathrm{MgO}}$. **a** The cross-sectional image obtained by transmission electron microscopy clearly shows the BLSO film, BaZrO_3_/MgO template bilayer, and Al_2_O_3_ substrate. **b** The fast Fourier transformation images at selected areas indicate the (111)-oriented epitaxial growth of the BLSO film and BaZrO_3_ template layer. **c** Energy-dispersive X-ray spectroscopy shows the negligible intermixing of Sn (blue colour), Zr (red), Mg (green), and Al (yellow) atoms between the layers

For additional insight into high-quality BLSO epitaxial films, cross-sectional images of ${\mathrm{BLSO}}_{(0001){\mathrm{Al}}_{2}{\mathrm{O}}_{3}}^{{\mathrm{BaZrO}}_{3}/\mathrm{MgO}}$ were acquired by TEM (Fig. 2a). The BLSO film, BaZrO_3_ layer, MgO layer, and Al_2_O_3_ substrate are distinguished by dark and bright regions arising from their different atomic numbers. The surface of (111)-oriented BLSO films exhibited triangular characteristics with {100} facets since most perovskite phases have the lowest energy surfaces among the {001} surfaces [27]. However, the surface atomic force microscopy image of the 350-nm-thick BLSO films revealed flatness over a 1 × 1-μm^2^ area with a low roughness of 2.3 ± 0.5 nm (Additional file 1: Fig. S2). Fast Fourier transformation of high-resolution TEM images also indicated the (111)-oriented epitaxial growth of the BLSO film and BaZrO_3_ template layer (Fig. 2b). Energy-dispersive X-ray spectroscopy indicated uniform distributions of Sn (blue), Zr (red), Mg (green), and Al (yellow) atoms over the entire area, indicating minimal atomic intermixing among the film, template layers, and substrate.

### Sheet resistance decreased by ~ 50 times in BLSO epitaxial films on (0001)Al_2_O_3_

**Fig. 3:**
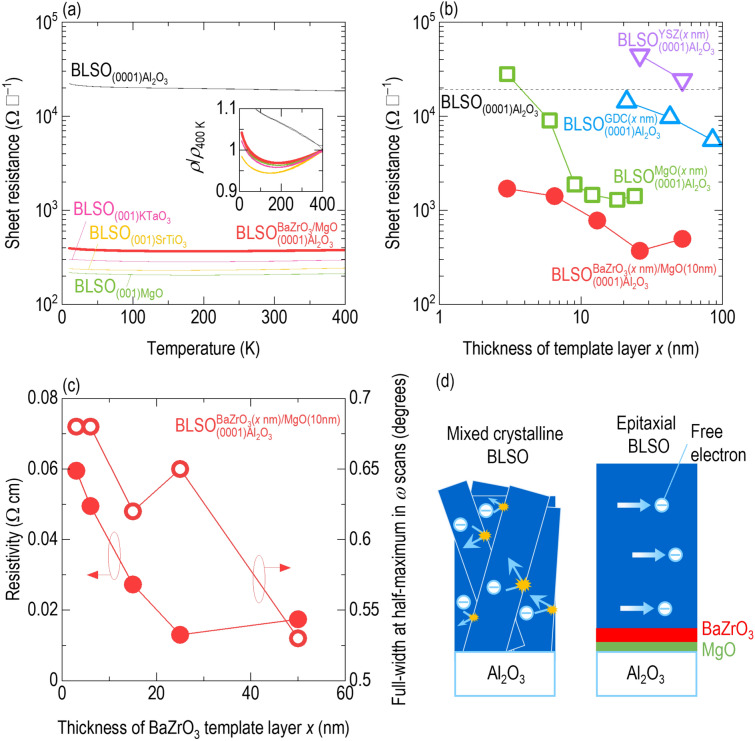
Single-crystalline-level conductive property of ${\mathrm{BLSO}}_{(0001){\mathrm{Al}}_{2}{\mathrm{O}}_{3}}^{{\mathrm{BaZrO}}_{3}/\mathrm{MgO}}$. **a** The sheet resistance of ${\mathrm{BLSO}}_{(0001){\mathrm{Al}}_{2}{\mathrm{O}}_{3}}^{{\mathrm{BaZrO}}_{3}/\mathrm{MgO}}$ is ~ 50 times lower than that of ${\mathrm{BLSO}}_{(0001){\mathrm{Al}}_{2}{\mathrm{O}}_{3}}$ and is comparable to those of single-crystalline BLSO epitaxial films grown on KTaO_3_, SrTiO_3_, and MgO. The inset shows that the resistivities *ρ*, normalized by the 400 K resistivity *ρ*_400 K_, of ${\mathrm{BLSO}}_{(0001){\mathrm{Al}}_{2}{\mathrm{O}}_{3}}^{{\mathrm{BaZrO}}_{3}/\mathrm{MgO}}$, ${\mathrm{BLSO}}_{(001){\mathrm{KTaO}}_{3}}$﻿, ${\mathrm{BLSO}}_{(001){\mathrm{SrTiO}}_{3}}$, and ${\mathrm{BLSO}}_{(001)\mathrm{MgO}}$ increase above 150 K, indicating metallicity near room temperature. **b** The sheet resistances of ${\mathrm{BLSO}}_{(0001){\mathrm{Al}}_{2}{\mathrm{O}}_{3}}^{{\mathrm{BaZrO}}_{3}/\mathrm{MgO}}$ and ${\mathrm{BLSO}}_{(0001){\mathrm{Al}}_{2}{\mathrm{O}}_{3}}^{\mathrm{MgO}}$ decrease with increasing template layer thickness and are saturated above 10–20-nm-thick template layers. The dashed line indicates the sheet resistance of ${\mathrm{BLSO}}_{(0001){\mathrm{Al}}_{2}{\mathrm{O}}_{3}}$. **c** The lower resistivity of ${\mathrm{BLSO}}_{(0001){\mathrm{Al}}_{2}{\mathrm{O}}_{3}}^{{\mathrm{BaZrO}}_{3}/\mathrm{MgO}}$ with a thicker BaZrO_3_ template layer is attributed to enhanced crystallinity, as evidenced by a smaller full-width at half-maximum. **d** Compared with ${\mathrm{BLSO}}_{(0001){\mathrm{Al}}_{2}{\mathrm{O}}_{3}}$, there are fewer scattering centres for free electrons in ${\mathrm{BLSO}}_{(0001){\mathrm{Al}}_{2}{\mathrm{O}}_{3}}^{{\mathrm{BaZrO}}_{3}/\mathrm{MgO}}$

Figure 3a presents the temperature dependence of the sheet resistances of ${\mathrm{BLSO}}_{(0001){\mathrm{Al}}_{2}{\mathrm{O}}_{3}}^{{\mathrm{BaZrO}}_{3}/\mathrm{MgO}}$ and ${\mathrm{BLSO}}_{{(0001)\mathrm{Al}}_{2}{\mathrm{O}}_{3}}$. These data were compared with the sheet resistances of single-crystalline BLSO epitaxial films grown on cubic KTaO_3_, SrTiO_3_, and MgO substrates to emphasize the affirmative effect of templated epitaxy (see Additional file 1: Fig. S3–S5a for the corresponding XRD *θ*–2*θ* scans, *ω* scans, and resistivities). First, it should be noted that ${\mathrm{BLSO}}_{(0001){\mathrm{Al}}_{2}{\mathrm{O}}_{3}}$ exhibited a much higher sheet resistance of 19,000 Ω﻿ ${\Box^-1}$ at room temperature when compared with 210–290 Ω ${\Box^-1}$ for ${\mathrm{BLSO}}_{(001){\mathrm{KTaO}}_{3}}$, ${\mathrm{BLSO}}_{(001){\mathrm{SrTiO}}_{3}}$, and ${\mathrm{BLSO}}_{(001)\mathrm{MgO}}$. The sheet resistance of insulating ${\mathrm{BLSO}}_{{(0001)\mathrm{Al}}_{2}{\mathrm{O}}_{3}}$ decreased with increasing temperature; this is clearly indicated by the normalized resistivity $\rho /{\rho }_{400\mathrm{ K}}$ (*ρ*_400 K_: resistivity at 400 K) shown in the inset. The high sheet resistance of ${\mathrm{BLSO}}_{(0001){\mathrm{Al}}_{2}{\mathrm{O}}_{3}}$ is equivalent to a resistivity of ~ 1 Ω cm, which is similar to the 1–10 Ω cm resistivity of polycrystals [8].

The BaZrO_3_/MgO template bilayer significantly recovered the conductive properties of BLSO epitaxial films on (0001)Al_2_O_3_ to the single-crystalline level. The sheet resistance of ${\mathrm{BLSO}}_{(0001){\mathrm{Al}}_{2}{\mathrm{O}}_{3}}^{{\mathrm{BaZrO}}_{3}/\mathrm{MgO}}$ was 370 Ω ^–1^ at room temperature, which was significantly lower by ~ 50 times than that of ${\mathrm{BLSO}}_{{(0001)\mathrm{Al}}_{2}{\mathrm{O}}_{3}}$ and comparable to those of single-crystalline ${\mathrm{BLSO}}_{(001){\mathrm{KTaO}}_{3}}$, ${\mathrm{BLSO}}_{(001){\mathrm{SrTiO}}_{3}}$, and ${\mathrm{BLSO}}_{(001)\mathrm{MgO}}$. Figure 3a and the corresponding inset show that the sheet resistance and normalized resistivity of ${\mathrm{BLSO}}_{(0001){\mathrm{Al}}_{2}{\mathrm{O}}_{3}}^{{\mathrm{BaZrO}}_{3}/\mathrm{MgO}}$ increased with increasing temperature, indicating metallic behaviour near room temperature. Insulating behaviour below 150 K was also observed for BLSO epitaxial films grown on cubic substrates. Hall measurements (Additional file 1: Fig. S5b) established that the carrier density and mobility of ${\mathrm{BLSO}}_{{(0001)\mathrm{Al}}_{2}{\mathrm{O}}_{3}}^{{\mathrm{BaZrO}}_{3}/\mathrm{MgO}}$ were 8.7 × 10^20^ cm^−3^ and 0.14 cm^2^ V^−1^ s^−1^, respectively, compared with 2.8–4.1 × 10^20^ cm^−3^ and 1.5–3.3 cm^2^ V^−1^ s^−1^ for single-crystalline ${\mathrm{BLSO}}_{(001){\mathrm{KTaO}}_{3}}$, ${\mathrm{BLSO}}_{(001){\mathrm{SrTiO}}_{3}}$, and ${\mathrm{BLSO}}_{(001)\mathrm{MgO}}$ (Additional file 1: Table S1).

The enhanced conductivity of BLSO epitaxial films on Al_2_O_3_ with the assistance of BaZrO_3_/MgO template bilayer motivated us to explore other template layers for growing conductive BLSO epitaxial films on Al_2_O_3_. The template layer should satisfy two important requirements, i.e., epitaxy and a flat surface, to ensure the growth of epitaxial BLSO films. (111)-Oriented YSZ, Gd-doped CeO_2_ (GDC), and MgO (without BaZrO_3_ layer) epitaxial films were grown on (0001)Al_2_O_3_ (Additional file 1: Fig. S6a–c) [20, 25, 28–32]. The MgO, YSZ, and GDC epitaxial films had very flat surfaces with a low roughness of 1.4−1.7 nm, as indicated by the clear X-ray reflectivity fringe patterns (Additional file 1: Fig. S7). While (111)-oriented BLSO films were epitaxially grown on a (111)MgO template layer (Additional file 1: Fig. S8a), (011)-oriented BLSO epitaxial films were grown on (111)YSZ (Additional file 1: Fig. S9a) and (111)GDC template layers (Additional file 1: Fig. S10a). Using XRD *θ–2θ* scans and *ϕ* scans, epitaxial relationships were established for (111)BLSO || (111)MgO || (001)Al_2_O_3_ and $[1\overline{1 }0]$ BLSO || $[1\overline{1 }0]$ MgO || [100]Al_2_O_3_ for ${\mathrm{BLSO}}_{(0001){\mathrm{Al}}_{2}{\mathrm{O}}_{3}}^{\mathrm{MgO}}$ (Additional file 1: Fig. S8b), (011)BLSO || (111)YSZ || (001)Al_2_O_3_ and $[100]$ BLSO || $[1\overline{1 }0]$ YSZ || [100]Al_2_O_3_ for ${\mathrm{BLSO}}_{(0001){\mathrm{Al}}_{2}{\mathrm{O}}_{3}}^{\mathrm{YSZ}}$ (Additional file 1: Fig. S9b), and (011)BLSO || (111)GDC || (001)Al_2_O_3_ and $[0\overline{1 }1]$ BLSO || $[\overline{1 }\overline{1 }2]$ GDC || [100]Al_2_O_3_ for ${\mathrm{BLSO}}_{(0001){\mathrm{Al}}_{2}{\mathrm{O}}_{3}}^{\mathrm{GDC}}$ (Additional file 1: Fig. S10b). Table 1 also includes details of the crystal structures of the MgO, YSZ, and GDC template layers, lattice parameters, epitaxial relationships among the BLSO film, template layer, and (0001)Al_2_O_3_ substrate, and in-plane lattice mismatch.

Among the template layers used in this work, the BaZrO_3_/MgO template bilayer provided the most conductive BLSO epitaxial films. Figure 3b shows that the sheet resistance of ${\mathrm{BLSO}}_{(0001){\mathrm{Al}}_{2}{\mathrm{O}}_{3}}^{{\mathrm{BaZrO}}_{3}/\mathrm{MgO}}$ reached 370 Ω ${\Box}$﻿^–1^ at room temperature, while ${\mathrm{BLSO}}_{(0001){\mathrm{Al}}_{2}{\mathrm{O}}_{3}}^{\mathrm{MgO}}$, ${\mathrm{BLSO}}_{(0001){\mathrm{Al}}_{2}{\mathrm{O}}_{3}}^{\mathrm{GDC}}$, and ${\mathrm{BLSO}}_{(0001){\mathrm{Al}}_{2}{\mathrm{O}}_{3}}^{\mathrm{YSZ}}$ had minimum sheet resistances of 1300, 5600, and 24,000 Ω ﻿${\Box}$^–1^, respectively. The lowest sheet resistance of ${\mathrm{BLSO}}_{(0001){\mathrm{Al}}_{2}{\mathrm{O}}_{3}}^{{\mathrm{BaZrO}}_{3}/\mathrm{MgO}}$ was consistent with the smallest FWHM of 0.39°, which is comparable to 1.09° for ${\mathrm{BLSO}}_{(0001){\mathrm{Al}}_{2}{\mathrm{O}}_{3}}^{\mathrm{MgO}}$ (Additional file 1: Fig. S8c), 1.04° for ${\mathrm{BLSO}}_{(0001){\mathrm{Al}}_{2}{\mathrm{O}}_{3}}^{\mathrm{YSZ}}$ (Additional file 1: Fig. S9c), and 3.12° for ${\mathrm{BLSO}}_{(0001){\mathrm{Al}}_{2}{\mathrm{O}}_{3}}^{\mathrm{GDC}}$ (Additional file 1: Fig. S10c). The other notable feature of Fig. 3b is that the sheet resistance of the BLSO films decreased with increasing template layer thickness (see Additional file 1: Fig. S11 for raw data.). The sheet resistances of the BLSO films on the MgO and BaZrO_3_/MgO (10 nm) template layers were saturated when the MgO or BaZrO_3_ layers were thicker than 10–20 nm. Figure 3c shows that the resistivity and FWHM for (222)BLSO of ${\mathrm{BLSO}}_{(0001){\mathrm{Al}}_{2}{\mathrm{O}}_{3}}^{{\mathrm{BaZrO}}_{3}/\mathrm{MgO}}$ tended to decrease with increasing BaZrO_3_ template layer thickness. This indicates that the crystallinity of the BLSO epitaxial films was enhanced for thicker template layers due to relaxation of substrate-induced strain. Although the sheet resistances also decreased with increasing YSZ and GDC template layer thickness, this was not further investigated due to the appearance of mixed phases in very thick YSZ (> 100 nm) and GDC (> 350 nm) films (Additional file 1: Fig. S6d and e).

We hypothesized that the ~ 50-times-higher conductive BLSO epitaxial films on (0001)Al_2_O_3_ might be attributed to the enhanced crystallinity promoted by the BaZrO_3_/MgO template bilayer. Figure 3d schematically illustrates the correlation between enhanced crystallinity and suppressed electron scattering. The decreased conduction of ${\mathrm{BLSO}}_{(0001){\mathrm{Al}}_{2}{\mathrm{O}}_{3}}$ might be due to electron scattering at the many grain boundaries of the mixed-crystalline films, as reported for polycrystalline specimens [8, 9]. On the other hand, there were few scattering centres in epitaxial ${\mathrm{BLSO}}_{(0001){\mathrm{Al}}_{2}{\mathrm{O}}_{3}}^{{\mathrm{BaZrO}}_{3}/\mathrm{MgO}}$ films, resulting in free electrons.

### High transmittance of BLSO epitaxial films on Al_2_O_3_

**Fig. 4:**
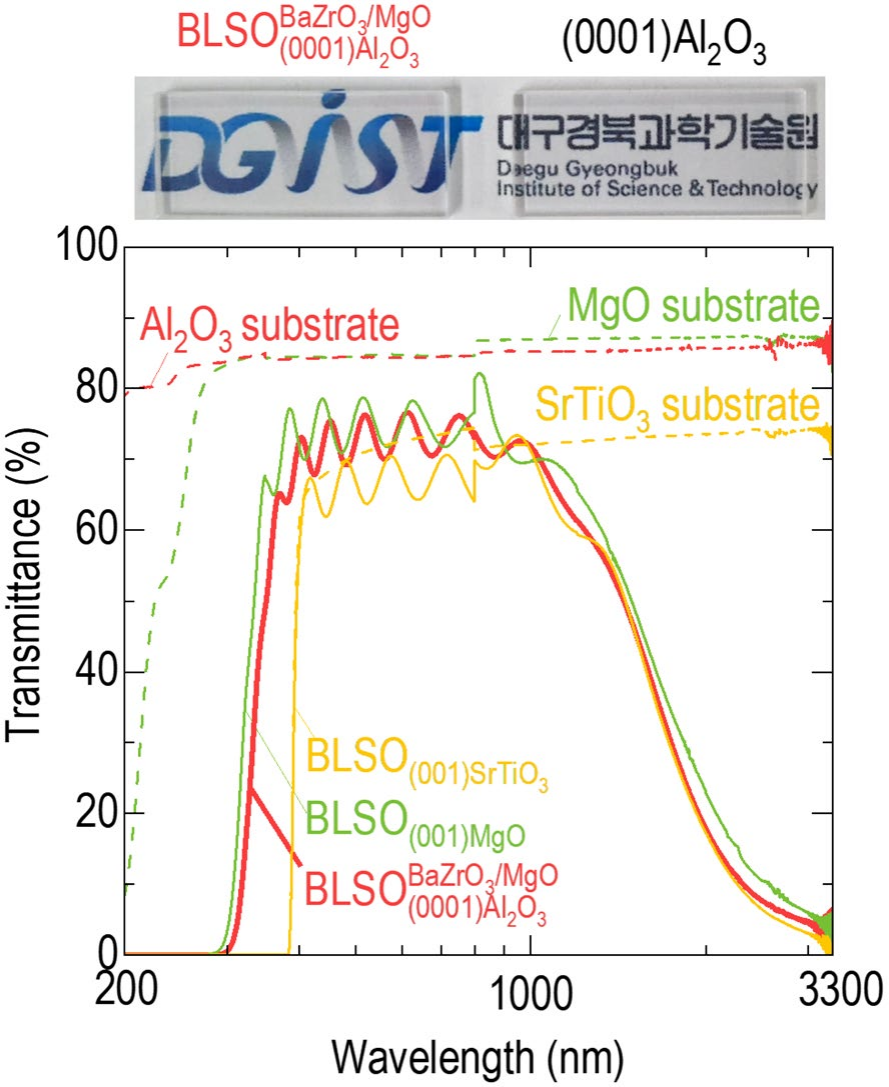
Transmittance of ${\mathrm{BLSO}}_{(0001){\mathrm{Al}}_{2}{\mathrm{O}}_{3}}^{{\mathrm{BaZrO}}_{3}/\mathrm{MgO}}$. The optical images on top of figure show that we can see a “DGIST” logo over film and bare Al_2_O_3_ substrate, indicating that the transparency of our ${\mathrm{BLSO}}_{(0001){\mathrm{Al}}_{2}{\mathrm{O}}_{3}}^{{\mathrm{BaZrO}}_{3}/\mathrm{MgO}}$ films is as high as that of the substrate. All BLSO epitaxial films show high transmittances (> 75%) in the visible region and suppressed transmittances in the infrared region. The fundamental absorption edges of ${\mathrm{BLSO}}_{(0001){\mathrm{Al}}_{2}{\mathrm{O}}_{3}}^{{\mathrm{BaZrO}}_{3}/\mathrm{MgO}}$ and ${\mathrm{BLSO}}_{(001)\mathrm{MgO}}$, at which the transmittance drops sharply at ultraviolet wavelengths, are located at shorter wavelengths than that of ${\mathrm{BLSO}}_{(001){\mathrm{SrTiO}}_{3}}$. The dashed lines indicate the transmittances of Al_2_O_3_, MgO, and SrTiO_3_ substrates

The very large bandgap (> 6.2 eV) of Al_2_O_3_ guarantees very high transmittance (~ 85% at 200–6000 nm) over a wide spectral range; this is not the case for SrTiO_3_ and KTaO_3_, which have a small bandgap of ~ 3.2 eV (~ 75% at 390–5000 nm). Therefore, Al_2_O_3_ enabled us to investigate the ultraviolet performance of BLSO, which was impossible for SrTiO_3_ and KTaO_3_. The solid lines in Fig. 4 show the transmittance of ${\mathrm{BLSO}}_{(0001){\mathrm{Al}}_{2}{\mathrm{O}}_{3}}^{{\mathrm{BaZrO}}_{3}/\mathrm{MgO}}$, ${\mathrm{BLSO}}_{(001){\mathrm{SrTiO}}_{3}}$, and ${\mathrm{BLSO}}_{(001)\mathrm{MgO}}$ in the wavelength range of 200–3300 nm. ${\mathrm{BLSO}}_{(0001){\mathrm{Al}}_{2}{\mathrm{O}}_{3}}^{{\mathrm{BaZrO}}_{3}/\mathrm{MgO}}$ and ${\mathrm{BLSO}}_{(001)\mathrm{MgO}}$ showed higher transmittances of ~ 75% than ~ 65% of ${\mathrm{BLSO}}_{(001){\mathrm{SrTiO}}_{3}}$ in the visible wavelength range of 400–1000 nm, consistent with the higher visible transmittances of Al_2_O_3_ and MgO compared with that of SrTiO_3_ (dashed lines). The optical image on top of Fig. 4 emphasized high visible transmittance of ${\mathrm{BLSO}}_{(0001){\mathrm{Al}}_{2}{\mathrm{O}}_{3}}^{{\mathrm{BaZrO}}_{3}/\mathrm{MgO}}$. We could see the clear “DGIST” logo over the film and Al_2_O_3_ substrate. The oscillating transmittance was likely due to the interference between reflected light from the film and substrate [33], indicating 350-nm-thick BLSO films. The transmittance was typically suppressed at infrared wavelengths (> 1000 nm) due to the free electron response. However, the fundamental absorption edges at ultraviolet wavelengths, at which transmittance dropped sharply, depended on the substrate. The ${\mathrm{BLSO}}_{(001){\mathrm{SrTiO}}_{3}}$ and SrTiO_3_ substrate showed zero transmittance below nearly the same wavelength of ~ 390 nm, which corresponded to the ~ 3.2 eV bandgap of SrTiO_3_. This observation indicated that the drastic reduction in the ultraviolet transmittance of ${\mathrm{BLSO}}_{(001){\mathrm{SrTiO}}_{3}}$ was not due to the intrinsic properties of BLSO but possibly to the interband absorption between the O 2*p* and Ti 3*d* orbitals of SrTiO_3_. ${\mathrm{BLSO}}_{(0001){\mathrm{Al}}_{2}{\mathrm{O}}_{3}}^{{\mathrm{BaZrO}}_{3}/\mathrm{MgO}}$ and ${\mathrm{BLSO}}_{(001)\mathrm{MgO}}$ were transparent at a shorter wavelength of ~ 300 nm. Since the Al_2_O_3_ and MgO were transparent below 300 nm, they did not contribute to the absorption edge of ${\mathrm{BLSO}}_{(0001){\mathrm{Al}}_{2}{\mathrm{O}}_{3}}^{{\mathrm{BaZrO}}_{3}/\mathrm{MgO}}$. Therefore, ~ 4.1 eV was estimated as the bandgap of the BLSO films, which is similar to that of single crystals [8].

### High electromagnetic shielding effectiveness of BLSO epitaxial films deposited on (0001)Al_2_O_3_

**Fig. 5:**
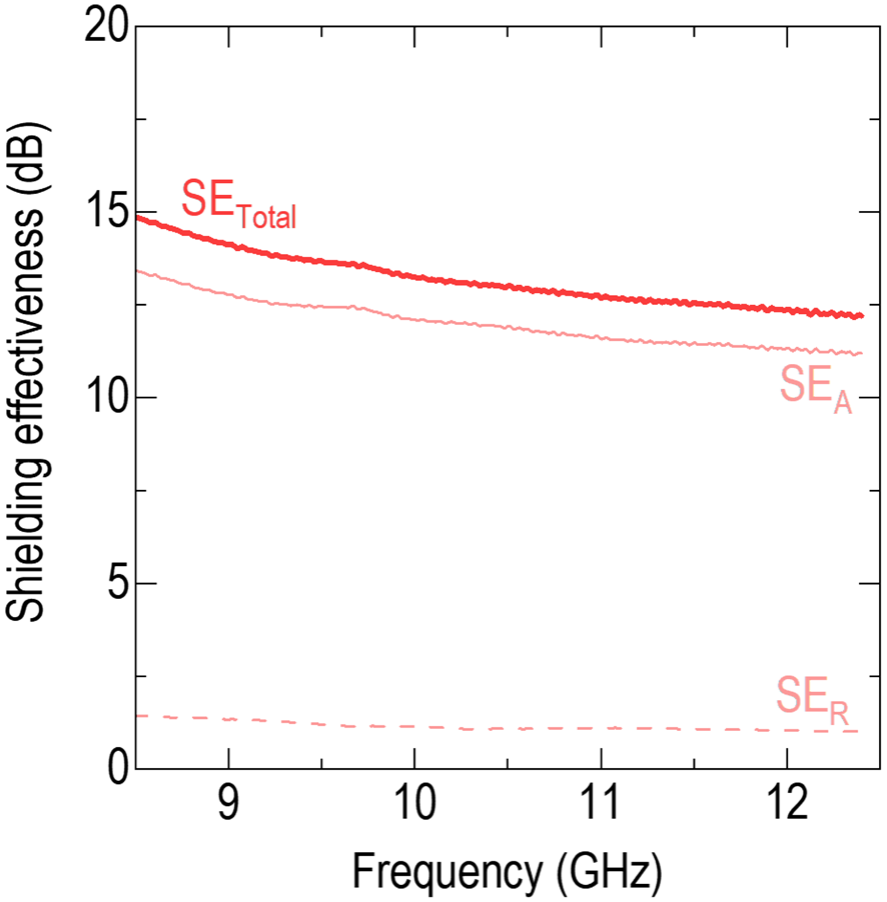
Total electromagnetic shielding effectiveness (SE) of ${\mathrm{BLSO}}_{(0001){\mathrm{Al}}_{2}{\mathrm{O}}_{3}}^{{\mathrm{BaZrO}}_{3}/\mathrm{MgO}}$. The films have an SE of ~ 13.2 dB at 10 GHz. SE_A_ and SE_R_ represent the transmitted wave through the films and Al_2_O_3_ substrate and the reflected wave from the BLSO film, respectively. The films have an SE_A_ of ~ 12.1 dB, which is larger than the SE_R_ of ~ 1.1 dB at 10 GHz

The high conductivity of BLSO provided profound electromagnetic shielding capability. We measured shielding effectiveness (SE) using the coaxial transmission line method. Figure 5 shows the SE of ${\mathrm{BLSO}}_{(0001){\mathrm{Al}}_{2}{\mathrm{O}}_{3}}^{{\mathrm{BaZrO}}_{3}/\mathrm{MgO}}$ in the X-band frequency range, i.e., 8.5–12.5 GHz, overlapped with radiowave (10^4^–10^10^ Hz) and microwave (10^9^–10^12^ Hz) ranges. The high SE of ~ 13.2 dB at 10 GHz was comparable to those of metal films, metal meshes, and two-dimensional materials (see Additional file 1: Table S2 for a comparison of resistivity, SE, and infrared transmittance among potential electromagnetic shielding materials) [34–41]. The SE (= SE_A_ + SE_R_) can be divided into SE_A_ and SE_R_, which denote shielding by absorption through ${\mathrm{BLSO}}_{(0001){\mathrm{Al}}_{2}{\mathrm{O}}_{3}}^{{\mathrm{BaZrO}}_{3}/\mathrm{MgO}}$ and reflection from the BLSO film, respectively. The larger SE_A_ of ~ 12.1 dB at 10 GHz than the SE_R_ of ~ 1.1 dB indicated that absorption was the dominant mechanism for electromagnetic shielding. Since the SE_A_ was higher than 10 dB, we ignored shielding by multiple reflections [35]. Thus, ${\mathrm{BLSO}}_{(0001){\mathrm{Al}}_{2}{\mathrm{O}}_{3}}^{{\mathrm{BaZrO}}_{3}/\mathrm{MgO}}$ is promising as a TC material for stealth technology, as also evidenced by its larger absorptivity (> 0.7 at 10 GHz) than reflectivity (< 0.2) (Additional file 1: Fig. S12). Our BLSO films will attract attention by virtue of having a low sheet resistance, high transmittance, high shielding effectiveness, chemical and mechanical stability, and easy coating fabrication process covering a wide range of applications.

### Thermal stability in air

**Fig. 6:**
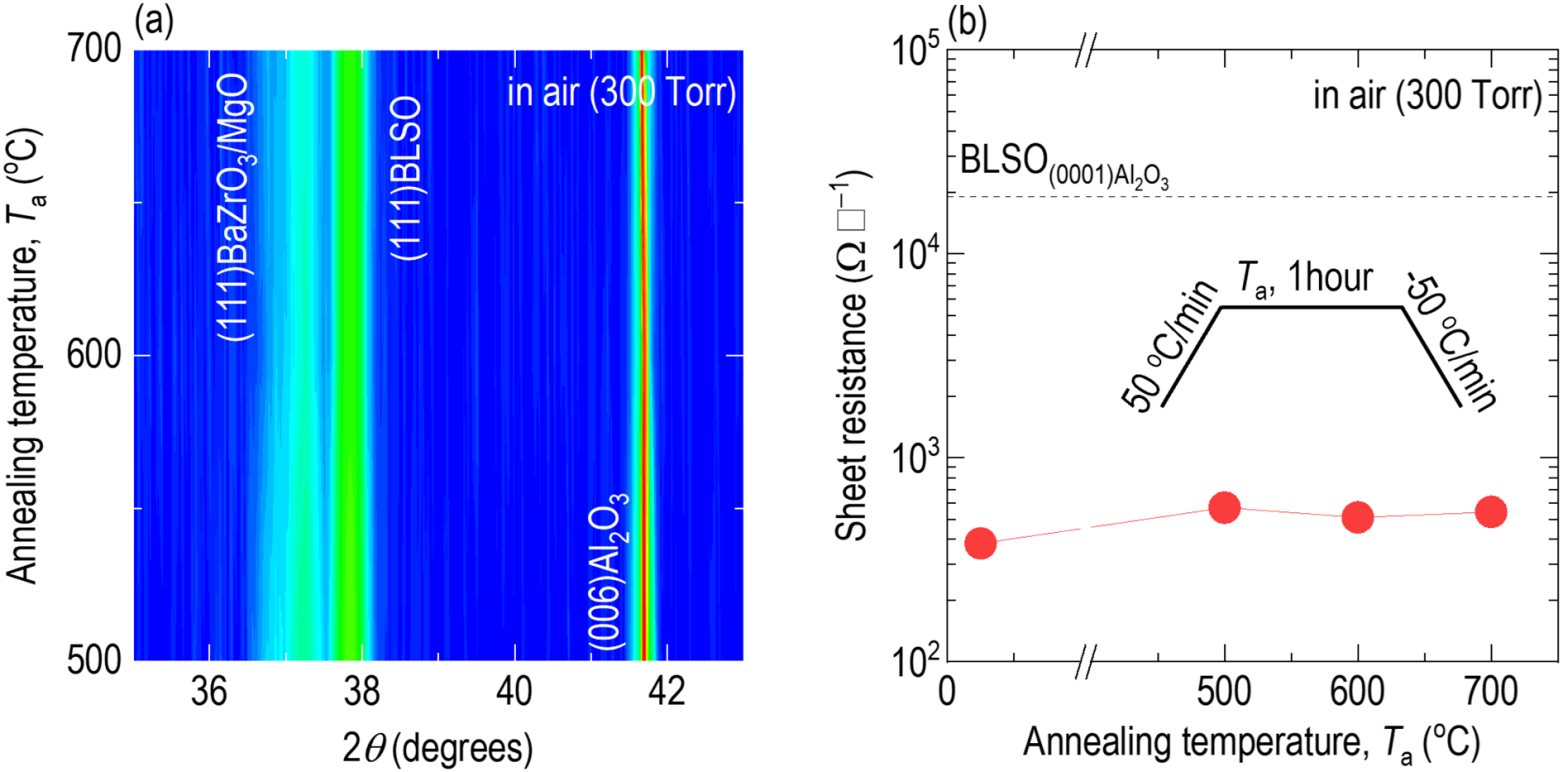
Thermal stability of ${\mathrm{BLSO}}_{(0001){\mathrm{Al}}_{2}{\mathrm{O}}_{3}}^{{\mathrm{BaZrO}}_{3}/\mathrm{MgO}}$. After annealing in air, even at 700 °C (inset), the **a** XRD *θ–2θ* scan and **b** sheet resistance of the as-grown films are persistent

To test the thermal stability, we increased the temperature at a rate of 50 °C per min, annealed the films at the annealing temperature (*T*_*a*_) in air for 1 h, and then decreased the temperature by 50 °C per min, as shown in the inset of Fig. 6b. The XRD *θ*-2*θ* scans revealed that the (111) diffraction peaks of BaZrO_3_/MgO and BLSO at 2*θ* = 37.2° and 37.8° were stable during annealing in air, even at 700 °C (Fig. 6a). The sheet resistance of ${\mathrm{BLSO}}_{(0001){\mathrm{Al}}_{2}{\mathrm{O}}_{3}}^{{\mathrm{BaZrO}}_{3}/\mathrm{MgO}}$ at room temperature also persisted (Fig. 6b) (see Additional file 1: Fig. S13 for raw data of the temperature dependence of sheet resistance). Thus, we found that the BaZrO_3_/MgO template bilayers did not deteriorate the thermal stability of BLSO. The thermal stability of ${\mathrm{BLSO}}_{(0001){\mathrm{Al}}_{2}{\mathrm{O}}_{3}}^{{\mathrm{BaZrO}}_{3}/\mathrm{MgO}}$ is sufficient for most applications below 700 °C.

## Conclusions

We achieved single-crystalline-level transparent conductive properties of BLSO epitaxial films on (0001)Al_2_O_3_ using BaZrO_3_/MgO template bilayer. The epitaxial films had a sheet resistance ~ 50 times lower than that of BLSO films directly grown on Al_2_O_3_. Using Al_2_O_3_ substrates guaranteed high ultraviolet transmittance (> 75%), which was rarely achieved in most previous studies of epitaxial BLSO films on expensive perovskites. The very conductive property guaranteed a high electromagnetic shielding effectiveness of ~ 13.2 dB at 10 GHz for the X-band. The phase was stable even at 700 °C in air. Due to their chemical/thermal/mechanical stability and economic benefits, the single-crystalline-level properties of BLSO films on Al_2_O_3_ would be suitable for applications, such as invisible circuitry, smart windows, and solar-energy harvesting, in extreme environments.

## Experimental section

*Templated epitaxy of La-doped BaSnO*_*3*_* (BLSO) epitaxial films* Using pulsed laser deposition, 350-nm-thick BLSO films were deposited on (0001)-oriented Al_2_O_3_ with BaZrO_3_/MgO template bilayer. To deposit the film and template layers, Ba_0.8_La_0.2_SnO_3_, BaZrO_3_, and MgO pellets were ablated using an excimer laser (IPEX-760; LightMachinery Inc.) with a wavelength of 248 nm, intensity of 1.5 J cm^–2^, and repetition rate of 10 Hz. The substrate was heated at 750 °C using a lamp heater. For BLSO and BaZrO_3_ deposition, an oxygen partial pressure of 75 mTorr was maintained by a mass flow controller. However, the diffraction peaks of MgO in the XRD *θ–2θ* scan disappeared when the MgO films were deposited at 75 mTorr, so 10 mTorr was used for MgO growth. For comparison, BLSO epitaxial films were deposited on various substrates with and without different template layers. The other substrates used in this research were $(1\overline{1 }02)$, $(11\overline{2 }0)$, $\mathrm{and} \left(10\overline{1 }0\right)$-oriented Al_2_O_3_, (001), (011), and (111)-oriented KTaO_3_, SrTiO_3_, MgO, and (111)-oriented YSZ. The template layers were MgO (without a BaZrO_3_ layer), YSZ, or GDC. YSZ and GDC template layers were deposited by ablating ZrO_2_-Y_2_O_3_ (8 mol%) and CeO_2_–Gd_2_O_3_ (10 mol%) pellets, respectively, under the same growth conditions for BLSO and BaZrO_3_. Since the electrical properties of BLSO significantly depended on the amount of doped La [8, 9], all films were grown using the same Ba_0.8_La_0.2_SnO_3_ pellet.

*Characterization of structural properties* Structural properties were investigated with a four-circle high-resolution X-ray diffractometer (Empyrean; PANalytical) that used Cu radiation with a wavelength of 1.54 Å. Cross-sectional images were acquired using a transmission electron microscope (HF-3300; Hitachi) at 300 kV with a lattice resolution of at least 1 Å. Fast Fourier transformation was performed using Digital Micrograph software (Gatan Inc.). Energy-dispersive X-ray spectroscopy was used to study the microstructure and elemental distribution in the film and template layers. An atomic force microscope (XE7; Park Systems) operating in tapping mode was used to obtain surface images and roughness values; the scan area and rate were 1 × 1 mm^2^ and 0.5 Hz, respectively.

*Measurement of transparent conductive properties* To investigate the transport properties, four Pt pads were deposited on the film surfaces by direct-current magnetron sputtering. Using a physical property measurement system (Quantum Design Inc.), the resistance (< 10 MΩ) was measured under an applied current upon cooling and subsequent heating over the temperature range of 10–400 K. The sheet resistance was calculated by multiplying the measured resistance by the geometric factor (2.5) of the films [42]. Hall measurements in a magnetic field ranging from − 4 to 4 T at 300 K were used to determine the carrier density and mobility. To directly measure transmittance, films were grown on double-sided polished substrates. Samples were also examined in the transmission mode of an ultraviolet–visible near-infrared spectrophotometer over the wavelength range of 175–3300 nm (Cary 5000; Agilent Technologies).

*Measurement of electromagnetic shielding effectiveness (SE)* Using a network analyser (N5222A; Agilent Technologies), we measured SE in a two-coaxial transmission line configuration. For this measurement, we grew the films on double-sided polished (0001)Al_2_O_3_ substrates (area: 22.8 × 10.1 mm^2^; thickness: 2 mm). The sample was positioned between two waveguides to measure the *S* parameters (*S*_11_ and *S*_21_) by emitting the electromagnetic wave from port 1. *S*_11_ was determined from the BLSO films by detecting the reflected wave at port 1. *S*_21_ was acquired by detecting the transmitted wave at port 2 through the films and Al_2_O_3_ substrate. The total SE was calculated by summing the contributions from absorption (${\mathrm{SE}}_{\mathrm{A}}=10\mathrm{log}\frac{1 - {\left|{S}_{11}\right|}^{2}}{{\left|{S}_{21}\right|}^{2}}$) and reflection (${\mathrm{SE}}_{\mathrm{R}}=10\mathrm{log}\frac{1}{1 - {\left|{S}_{11}\right|}^{2}}$) [35, 37]. We also calculated reflectivity $R (={\left|{S}_{11}\right|}^{2})$, transmissivity $T (={\left|{S}_{21}\right|}^{2})$, and absorptivity $A (=1-R-T$) [37].

## Supplementary Information

**Additional file 1.** Additional materials, additional figures S1–S13, additional tables S1, S2.

## Data Availability

The data supporting the findings of this study are available from the corresponding author upon reasonable request.
