# Supplementary material for: Strong conductivity enhancement of La-doped BaSnO3 transparent films on Al2O3 with the assistance of templated epitaxy for electromagnetic shielding in extreme environments
Source: Nano Converg. 2023 Feb 15;10:9. doi: 10.1186/s40580-023-00355-9 (PMC9931985; doi:10.1186/s40580-023-00355-9)

Additional file 1

Strong conductivity enhancement of La-doped BaSnO_3_ transparent films on Al_2_O_3_ with the assistance of templated epitaxy for electromagnetic shielding in extreme environments

Youngkyoung Ha and Shinbuhm Lee*

Department of Physics and Chemistry, Department of Emerging Materials Science, DGIST, Daegu 42988, Republic of Korea

*Corresponding author: Shinbuhm Lee

E-mail address: lee.shinbuhm@dgist.ac.kr

Keywords: Transparent conductors, La-doped BaSnO_3_, templated epitaxy, Al_2_O_3_, single-crystalline films

Figures S1−S13

Tables S1, S2

**1. Mixed-crystalline phases of La-doped BaSnO_3_ (BLSO) films directly grown on Al_2_O_3_**

We investigated whether La-doped BaSnO_3_ (BLSO) epitaxial films could be grown directly on Al_2_O_3_ without a template layer. Figure 1a shows that (0001)-oriented Al_2_O_3_ yielded non-epitaxial BLSO films. Figure S1a−c show X-ray diffraction (XRD) *θ*−2*θ* scans of BLSO films directly grown on $(1\bar{1}02)$, $(11\bar{2}0)$, and $\left( 10\bar{1}0 \right)$Al_2_O_3_, respectively. Despite very thick films of ~350 nm, there were very weak diffraction peaks of various crystallographic orientations. The direct growth of BLSO films on Al_2_O_3_ resulted in poor crystallinity overall. Hereafter, the simpler form of $\mathrm{BLSO}_{\mathrm{substrate}}^{template layers}$ will be used for convenience. For example, $\mathrm{BLSO}_{(0001)\mathrm{Al}_{2}O_{3}}^{\mathrm{BaZr}O_{3}/MgO}$ denotes BLSO epitaxial films grown on (0001)Al_2_O_3_ with BaZrO_3_/MgO template bilayer.


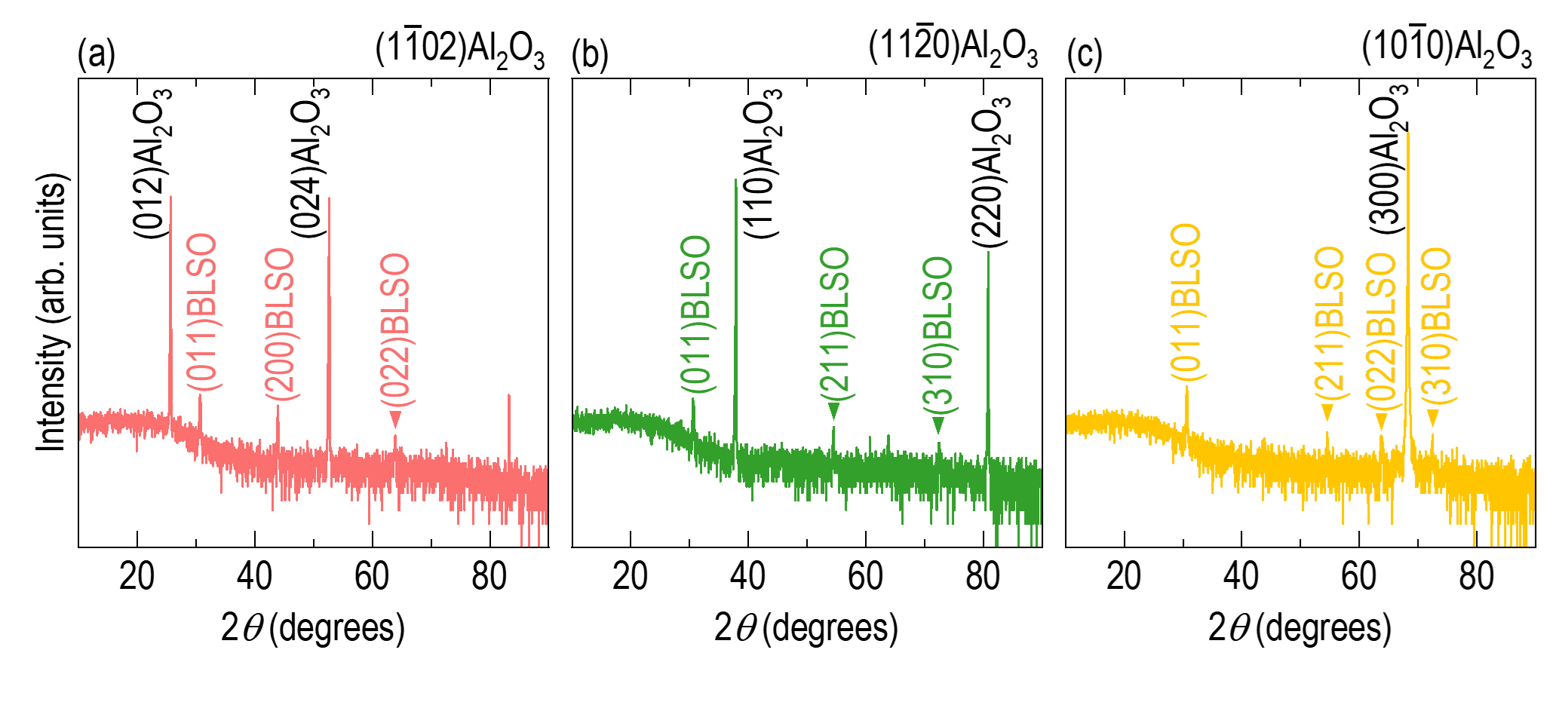


**Fig. S1.** X-ray diffraction *θ*−2*θ* scans of La-doped BaSnO_3_ (BLSO) films on (a) $(1\bar{1}02)$, (b) $(11\bar{2}0)$, and (c) $\left( 10\bar{1}0 \right)$-oriented Al_2_O_3_.

**2.** **Surface image and roughness of** $\mathbf{BLSO}_{\mathbf{(0001)}\mathbf{Al}_{\mathbf{2}}\mathbf{O}_{\mathbf{3}}}^{\mathbf{BaZr}\mathbf{O}_{\boldsymbol{3}}\mathbf{/MgO}}$

**
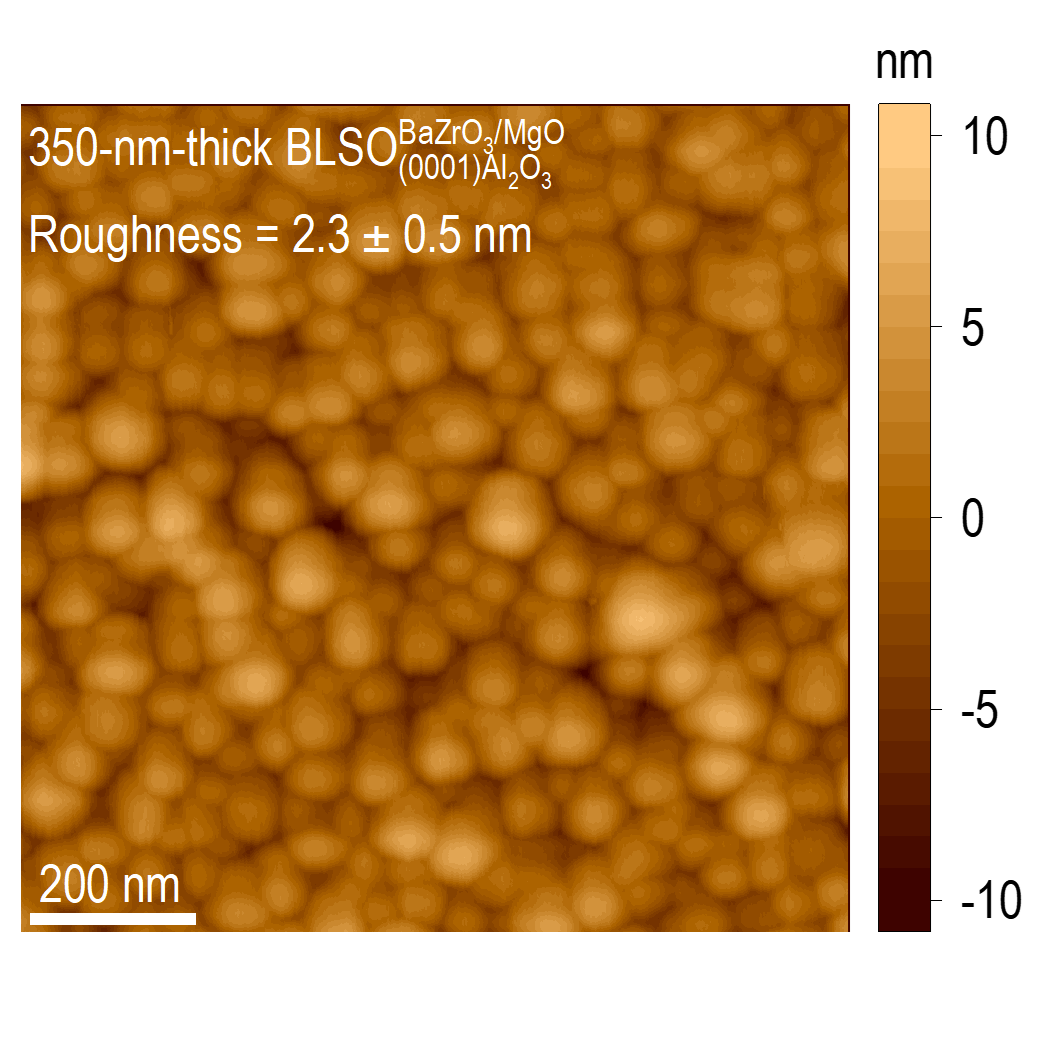
**

**Fig. S2.** Surface image of 350-nm-thick $\mathrm{BLSO}_{(0001)\mathrm{Al}_{2}O_{3}}^{\mathrm{BaZr}O_{3}/MgO}$ in a 1 × 1-*μ*m^2^-area, acquired by atomic force microscopy. Despite its thickness, the film surface has a very small roughness of 2.3 ± 0.5 nm.

**3. BLSO epitaxial films directly grown on cubic substrates**


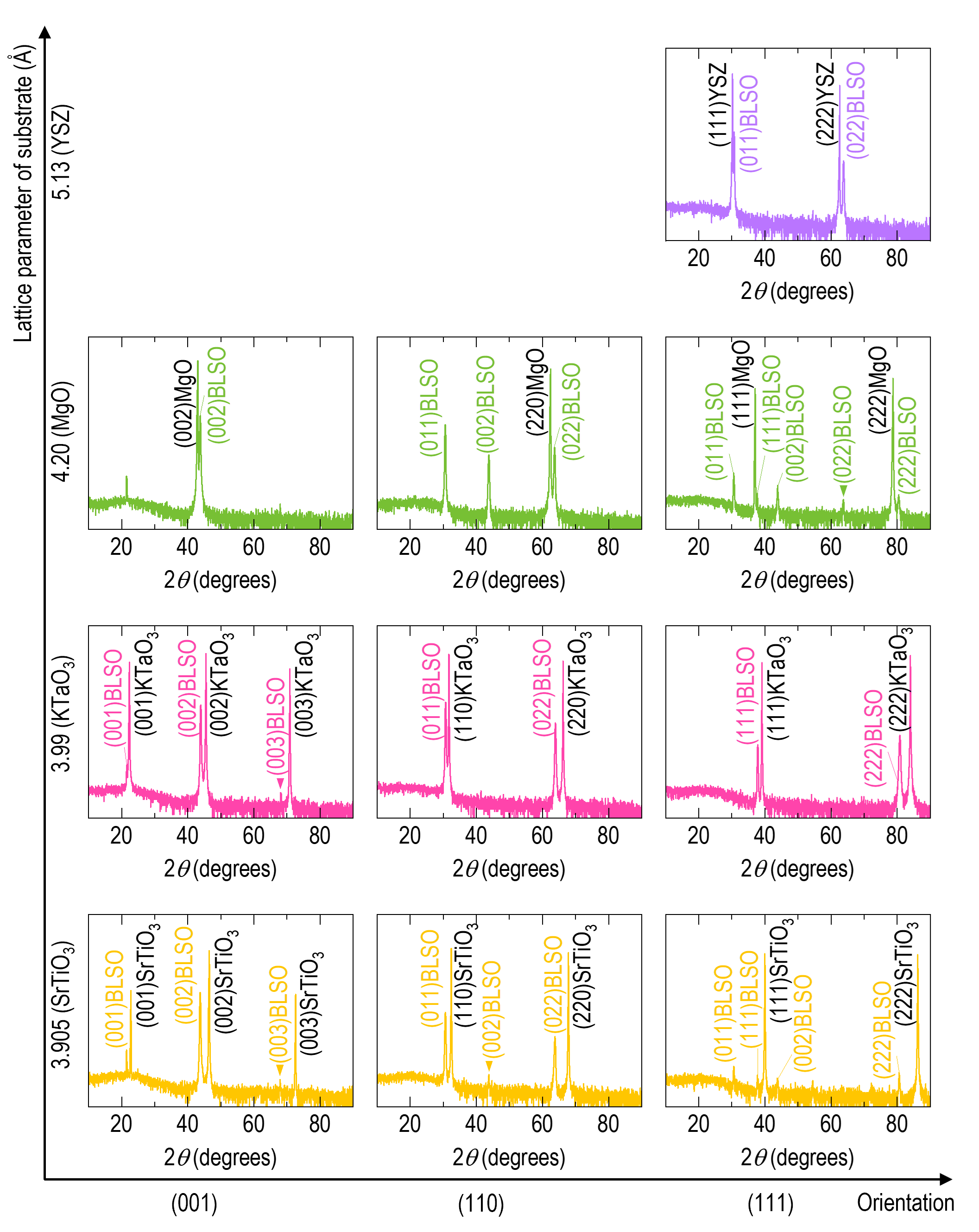


**Fig. S3.** Epitaxial stabilization of BLSO films on cubic substrates with various lattice parameters [Y-stabilized ZrO_2_ (YSZ), MgO, KTaO_3_, and SrTiO_3_] and crystal orientations [(001), (011), and (111)]. The XRD *θ*−2*θ* scans indicate that the growth of BLSO epitaxial films is attainable on (111)-oriented YSZ, (001)MgO, (001), (011), and (111)KTaO_3_, and (001)SrTiO_3_. The thickness of the BLSO films is 350 nm for all samples.


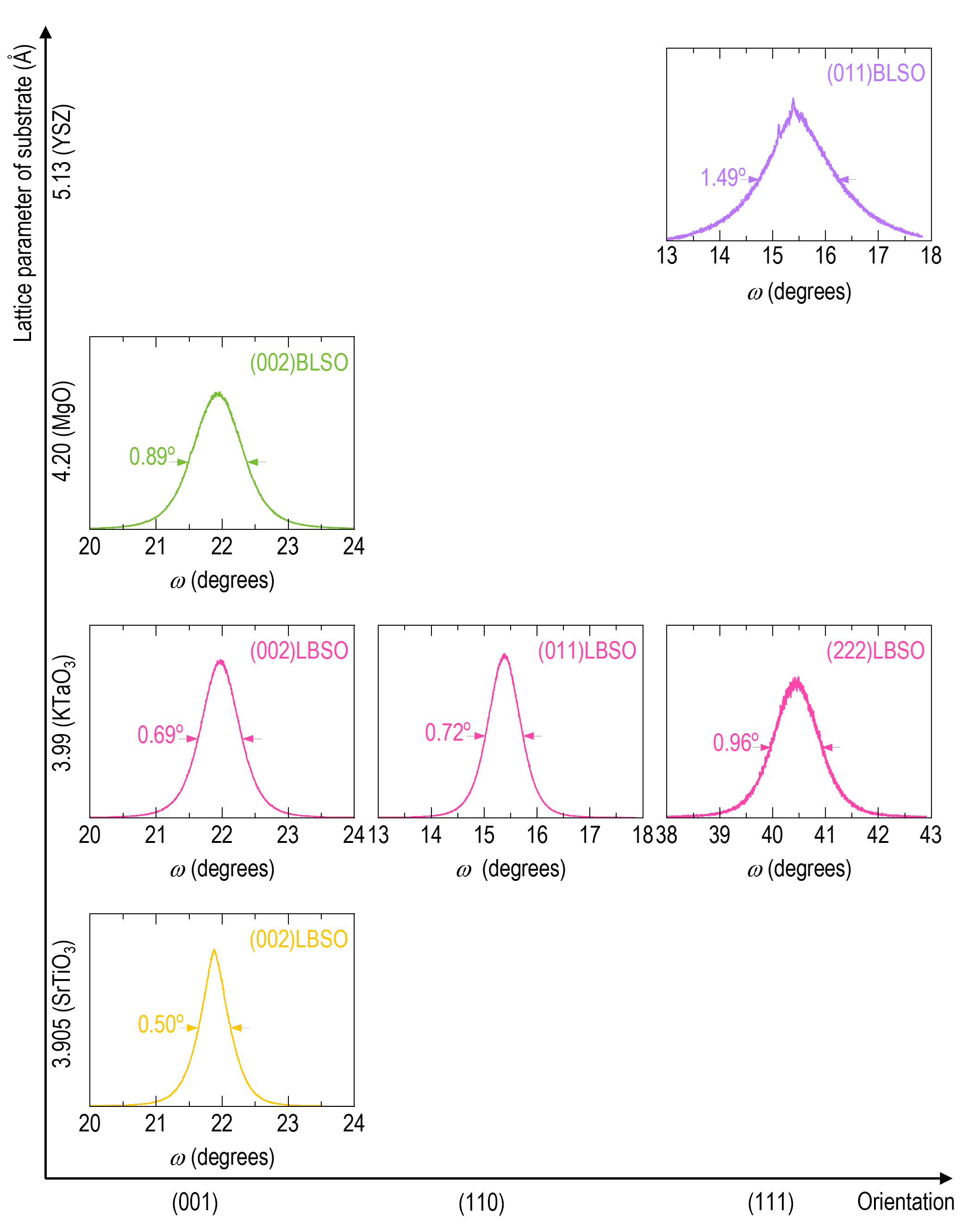


**Fig. S4.** X-ray diffraction *ω* scans of BLSO epitaxial films directly grown on cubic substrates. The rows and columns are the same as those in Fig. S3.

**4. Resistivity, carrier density, and mobility of BLSO epitaxial films**

We investigated the resistivity, carrier density, and mobility of BLSO epitaxial films grown on various substrates. Figure S5a shows the temperature dependence of the resistivity. The resistivity *ρ* was calculated according to the relationship $\rho=R_{S}W$, where *R*_S_ denotes sheet resistance and *W* is the BLSO film thickness of ~350 nm. The Hall coefficient was used to calculate the carrier density *n* at 300 K. The dependence of the Hall resistance on the magnetic field was quantified by sweeping the magnetic field strength from −4 to 4 T (Fig. S5b). The slope of the Hall resistance–magnetic field curve corresponds to the Hall coefficient. The carrier density was calculated by dividing the Hall coefficient by the elementary charge. Finally, the mobility was obtained by dividing the conductivity by the carrier density and elementary charge.


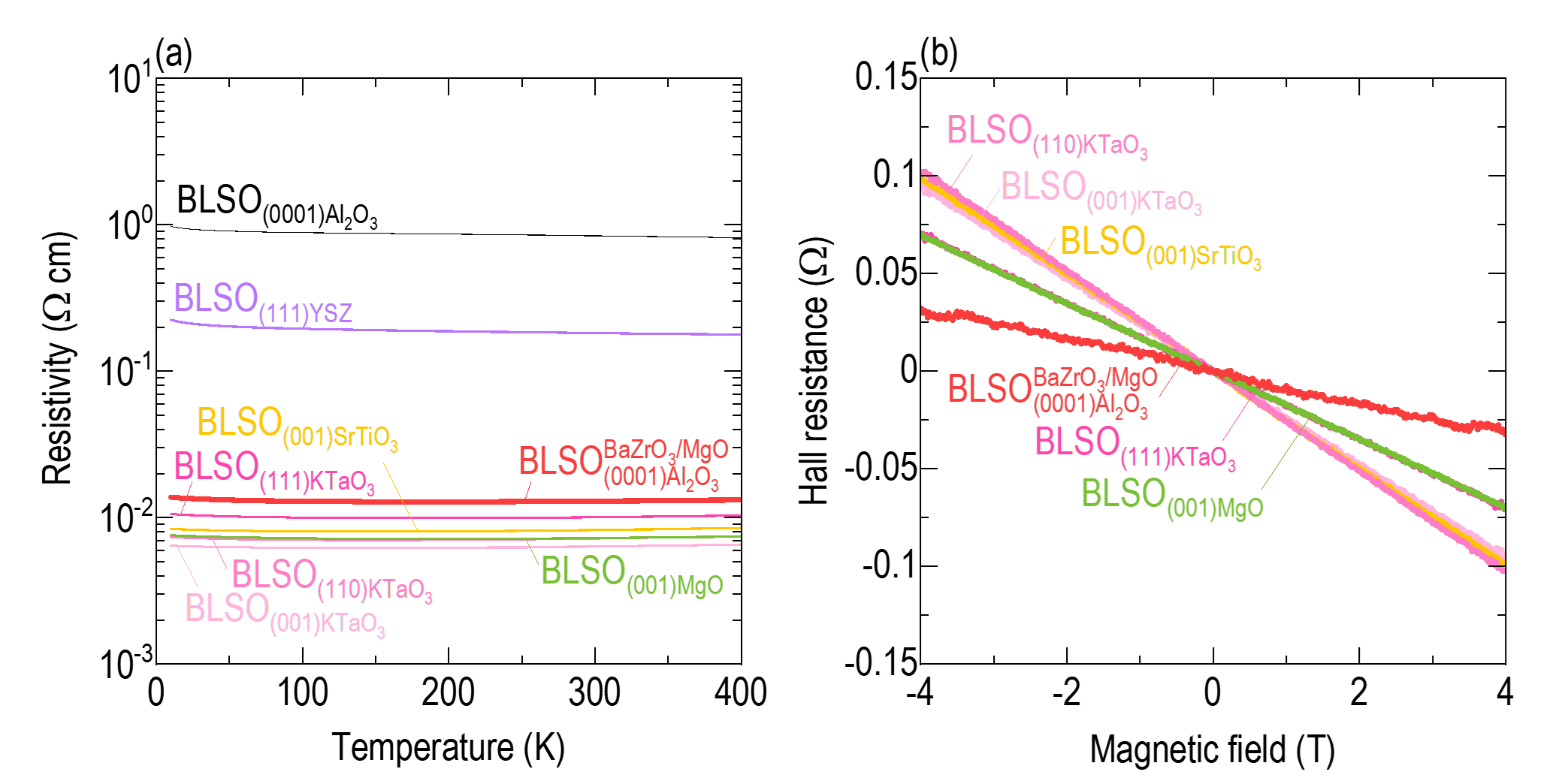


**Fig. S5.** Transport properties of BLSO epitaxial films. (a) Temperature dependence of resistivity. Compared with the high resistivity observed for mixed-crystalline $\mathrm{BLSO}_{\left( 0001 \right)\mathrm{Al}_{2}O_{3}}$, most BLSO epitaxial films have a low resistivity of ~10^−2^ Ω cm at room temperature. Although BLSO epitaxial films were grown on (111)YSZ, the $\mathrm{BLSO}_{\left( 111 \right)\mathrm{YSZ}}$ had a higher resistivity of ~1 Ω cm, consistent with the very high resistivity of $\mathrm{BLSO}_{(0001)\mathrm{Al}_{2}O_{3}}^{\mathrm{YSZ}}$. (b) Magnetic field dependence of Hall resistance. The negative slopes indicate that the BLSO epitaxial films have *n*-type semiconducting properties.

**Table S1.** Resistivity, carrier density, and mobility of BLSO epitaxial films.

| Template layer/substrate | Resistivity  (Ω cm) | Carrier density  (cm^−3^) | Mobility  (cm^2^ V^−1^ s^−1^) |
| --- | --- | --- | --- |
| BaZrO_3_/MgO/(0001)Al_2_O_3_ | 0.013 | 8.7 × 10^20^ | 0.14 |
| (001)KTaO_3_ | 0.0064 | 3.1 × 10^20^ | 3.3 |
| (110)KTaO_3_ | 0.0072 | 2.8 × 10^20^ | 3.1 |
| (111)KTaO_3_ | 0.011 | 4.1 × 10^20^ | 1.5 |
| (001)SrTiO_3_ | 0.0083 | 2.9 × 10^20^ | 2.6 |
| (001)MgO | 0.0073 | 4.1 × 10^20^ | 2.1 |

**5. Feasibility of MgO, Y-stabilized ZrO_2_ (YSZ), and Gd-doped CeO_2_ (GDC) template layers**

The template layer grown on (0001)-oriented Al_2_O_3_ should be an epitaxial film with a flat surface to ensure epitaxial growth of a BLSO film on the template layers.

**Epitaxial film:** The XRD *θ*−2*θ* scans in Fig. S6a−c revealed the epitaxial growth of MgO-, YSZ-, and Gd-doped CeO_2_ (GDC) template layers on (0001)-oriented Al_2_O_3_. There are two peaks at 2*θ* = 36.9° and 78.6° for MgO, 30.1° and 62.4° for YSZ, and 28.5° and 59.1° for GDC, corresponding to diffraction from the (111) and (222) planes, respectively. Except for the peaks at 2*θ* = 20.5°, 41.7°, and 64.5° generated by diffraction from the (003), (006), and (009) planes of Al_2_O_3_, no additional peaks were detected, which indicated the high quality of the MgO, YSZ, and GDC epitaxial films. Although very thick YSZ (> 100 nm) and GDC (> 350 nm) films exhibited mixed-crystalline phases (Fig. S6d and e), thin epitaxial films allowed us to use MgO, YSZ, and GDC for the template layer.

**Flat surface:** The X-ray reflectivity of MgO, YSZ, and GDC epitaxial films showed clear fringe patterns (Fig. S7), indicating very flat surfaces. By fitting the fringe patterns using the X’Pert Reflectivity programme, film thicknesses of 20 ± 1.7, 50 ± 1.4, and 85 ± 1.5 nm were obtained for the MgO, YSZ, and GDC epitaxial films, respectively. Film thickness was controlled by counting the number of excimer laser pulses during pulsed laser deposition. The growth rates were ~0.005, ~0.05, and ~0.2 Å per pulse for the MgO, YSZ, and GDC epitaxial films, respectively.


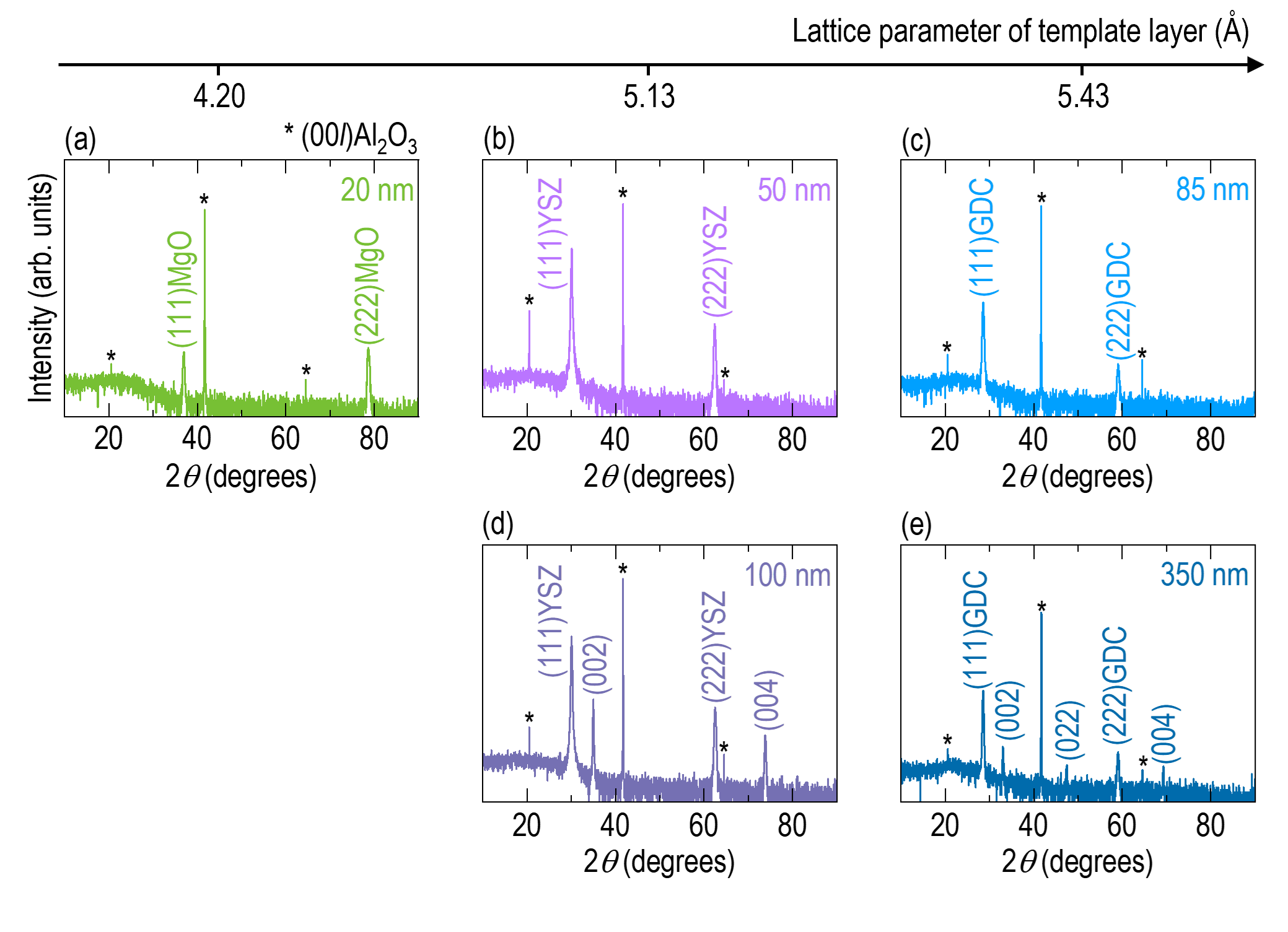


**Fig. S6.** X-ray diffraction *θ*−2*θ* scans of (a) 20-nm-thick MgO, (b) 50-nm-thick YSZ, (c) 85-nm-thick GDC, (d) 100-nm-thick YSZ, and (e) 350-nm-thick GDC films. The epitaxial growth of the MgO, YSZ, and GDC thin films was successful, although mixed-crystalline phases are apparent in the thick YSZ and GDC films. Since the thinner film is better for template layers, MgO, YSZ, and GDC can serve as template layers on which BLSO epitaxial films can be grown. The asterisks indicate the (003), (006), and (009) diffraction peaks of Al_2_O_3_.


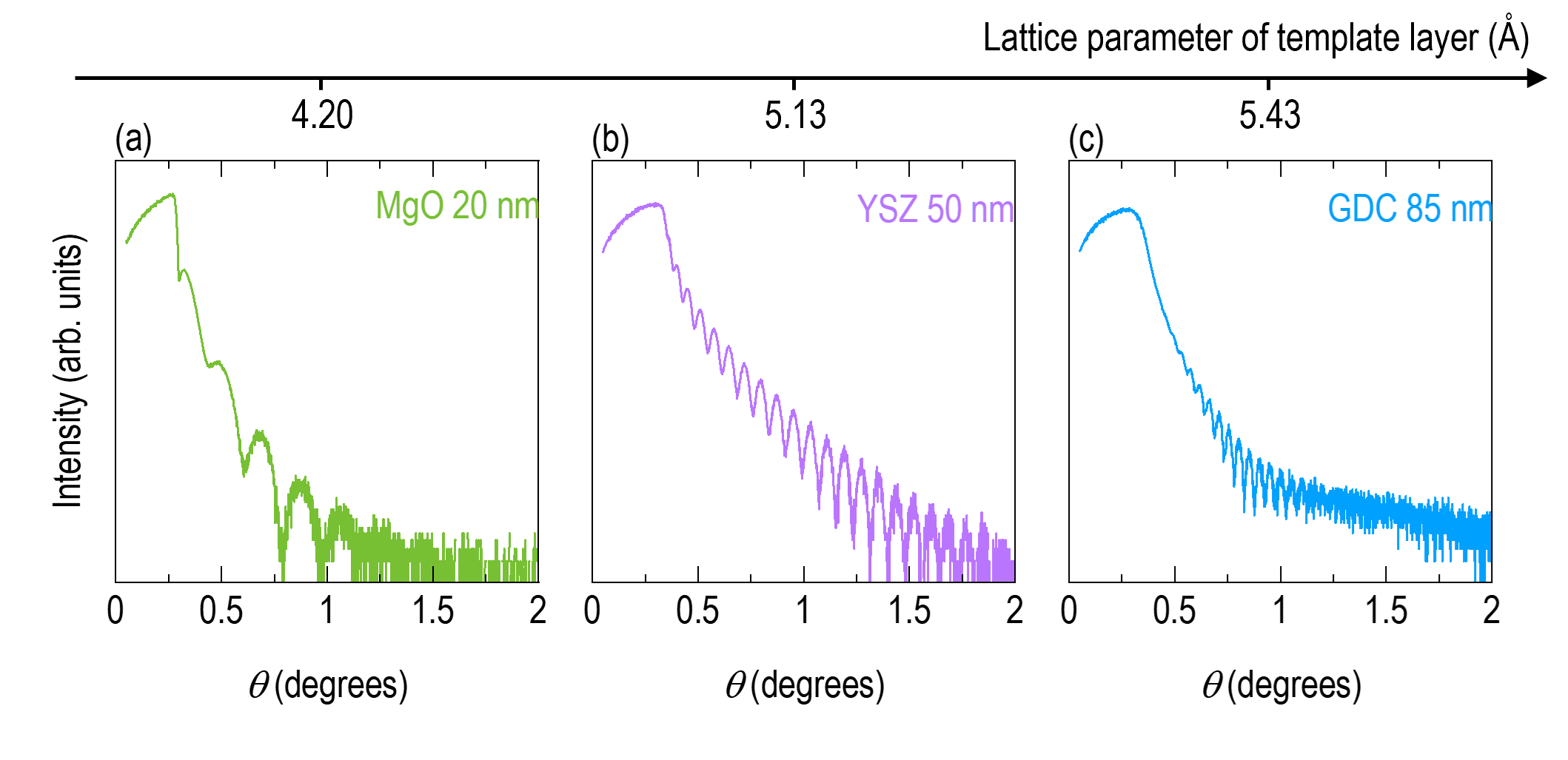


**Fig. S7.** X-ray reflectivity of (a) 20-nm-thick MgO, (b) 50-nm-thick YSZ, and (c) 85-nm-thick GDC films.

**6. Epitaxial stabilization of BLSO films on (0001)Al_2_O_3_ with MgO, YSZ, and GDC template layers**

We grew BLSO epitaxial films on (0001)Al_2_O_3_ using an MgO template layer. Figure S8a shows the XRD *θ*−2*θ* scan of $\mathrm{BLSO}_{(0001)\mathrm{Al}_{2}O_{3}}^{\mathrm{MgO}}$. In addition to the (111) and (222) diffraction peaks of the MgO template layer and the (006)Al_2_O_3_ diffraction peak (see Fig. S6a for details), two diffraction peaks were observed at 2*θ* = 37.8° and 80.8°, which were generated by diffraction from the (111) and (222) planes of the BLSO films. Using the XRD *φ* scans shown in Fig. S8b, epitaxial relationships were identified among the BLSO film, MgO template layer, and (0001)Al_2_O_3_ substrate. The {110}BLSO film and {220}MgO template layer showed six strong diffraction peaks that were separated by 30° from the {012}Al_2_O_3_ peaks. BLSO and MgO have cubic structures with similar lattice parameters, and (111)-oriented MgO and hexagonal Al_2_O_3_ have threefold symmetry about the axis perpendicular to the plane. Accordingly, the following epitaxial relationships were inferred, $[1\bar{1}0]$BLSO || $[1\bar{1}0]$MgO || [100]Al_2_O_3_ and (111)BLSO || (111)MgO || (001)Al_2_O_3_, as shown in the schematic illustrations. Figure S8c shows the XRD *ω* scan of $\mathrm{BLSO}_{(0001)\mathrm{Al}_{2}O_{3}}^{\mathrm{MgO}}$. The FWHM was 1.09° for (222)BLSO and 0.26° for (222)MgO.

We grew BLSO epitaxial films on (0001)Al_2_O_3_ using a YSZ template layer. Compared with $\mathrm{BLSO}_{(0001)\mathrm{Al}_{2}O_{3}}^{\mathrm{MgO}}$, the only different aspect of $\mathrm{BLSO}_{(0001)\mathrm{Al}_{2}O_{3}}^{\mathrm{YSZ}}$ was the formation of (011)-oriented BLSO epitaxial films. Figure S9a presents the XRD *θ*−2*θ* scan showing diffraction peaks corresponding to (011) and (022)BSLO at 2*θ* = 30.7° and 63.9°, respectively. The *φ* scans of the {222}BLSO, {220}YSZ, and {012}Al_2_O_3_ diffraction peaks of $\mathrm{BLSO}_{(0001)\mathrm{Al}_{2}O_{3}}^{\mathrm{YSZ}}$ were similar to those of $\mathrm{BLSO}_{(0001)\mathrm{Al}_{2}O_{3}}^{\mathrm{MgO}}$ (Fig. S8b). This indicated the epitaxial relationships of (011)BLSO || (111)YSZ || (001)Al_2_O_3_ and $[100]$BLSO || $[1\bar{1}0]$YSZ || [100]Al_2_O_3_, as shown in the schematic illustrations. The FWHM was 1.04° for (110)BLSO and 0.12° for (111)YSZ (Fig. S9c).

The GDC template layer also facilitated the (011)-oriented epitaxial growth of BLSO films on (0001)Al_2_O_3_, as shown by the XRD *θ*−2*θ* scan in Fig. S10a. However, different from those of $\mathrm{BLSO}_{(0001)\mathrm{Al}_{2}O_{3}}^{\mathrm{MgO}}$ and $\mathrm{BLSO}_{(0001)\mathrm{Al}_{2}O_{3}}^{\mathrm{YSZ}}$, the *φ* scans of {222}BLSO and {220}GDC showed threefold symmetric diffraction peaks at the same *φ*-angle of the substrate (Fig. S10b). This indicated the epitaxial relationships of (011)BLSO || (111)GDC || (001)Al_2_O_3_ and $[0\bar{1}1]$BLSO || $[\bar{1}\bar{1}2]$GDC || [100]Al_2_O_3_, as shown in the schematic illustrations. The FWHMs were quite large, i.e., 3.12° for (110)BLSO and 3.88° for (111)GDC (Fig. S10c).

**
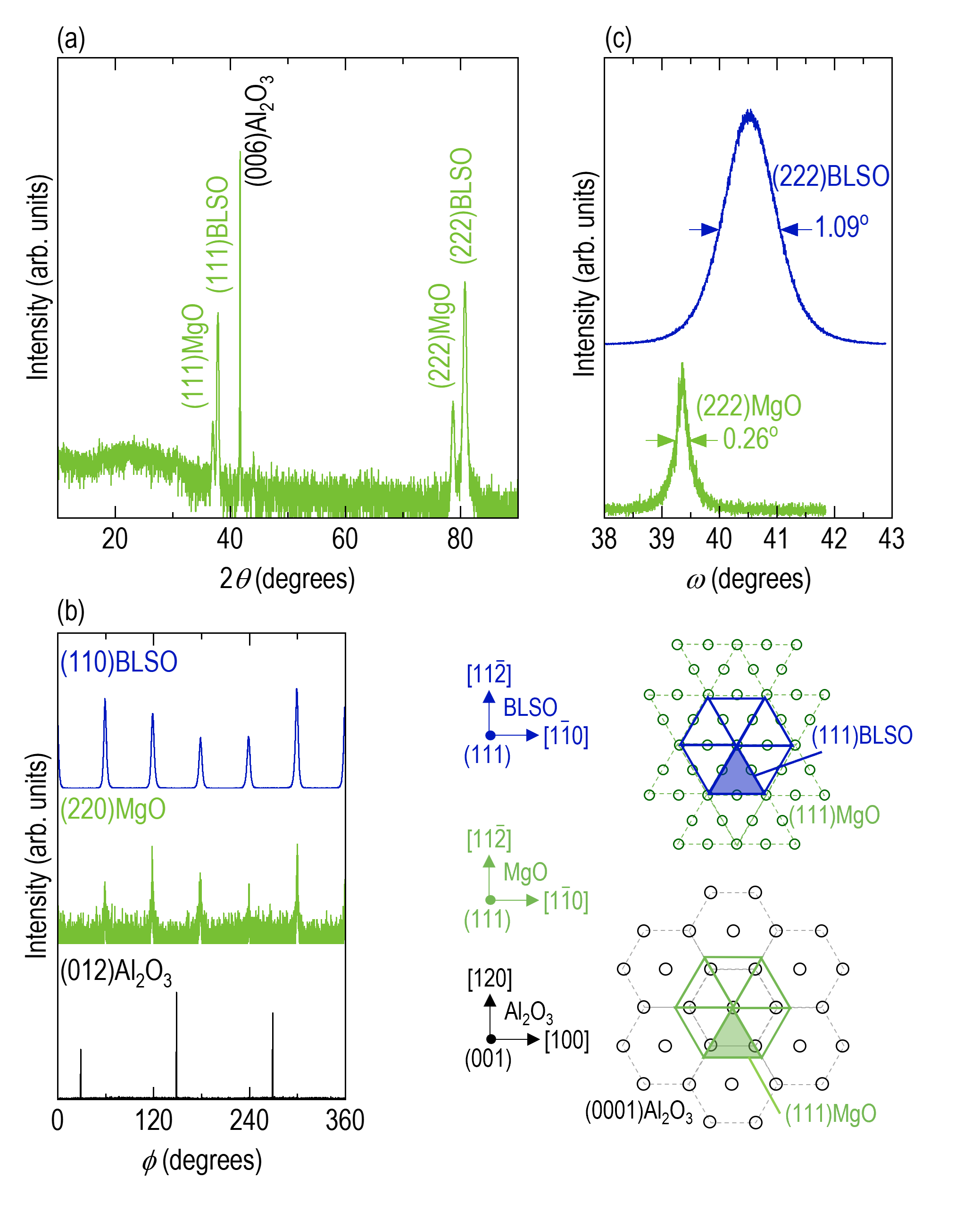
**

**Fig. S8.** (a) X-ray diffraction *θ*−2*θ* scan, (b) *φ* scan and in-plane matching, and (c) *ω* scan of $\mathrm{BLSO}_{(0001)\mathrm{Al}_{2}O_{3}}^{\mathrm{MgO}}$.

**
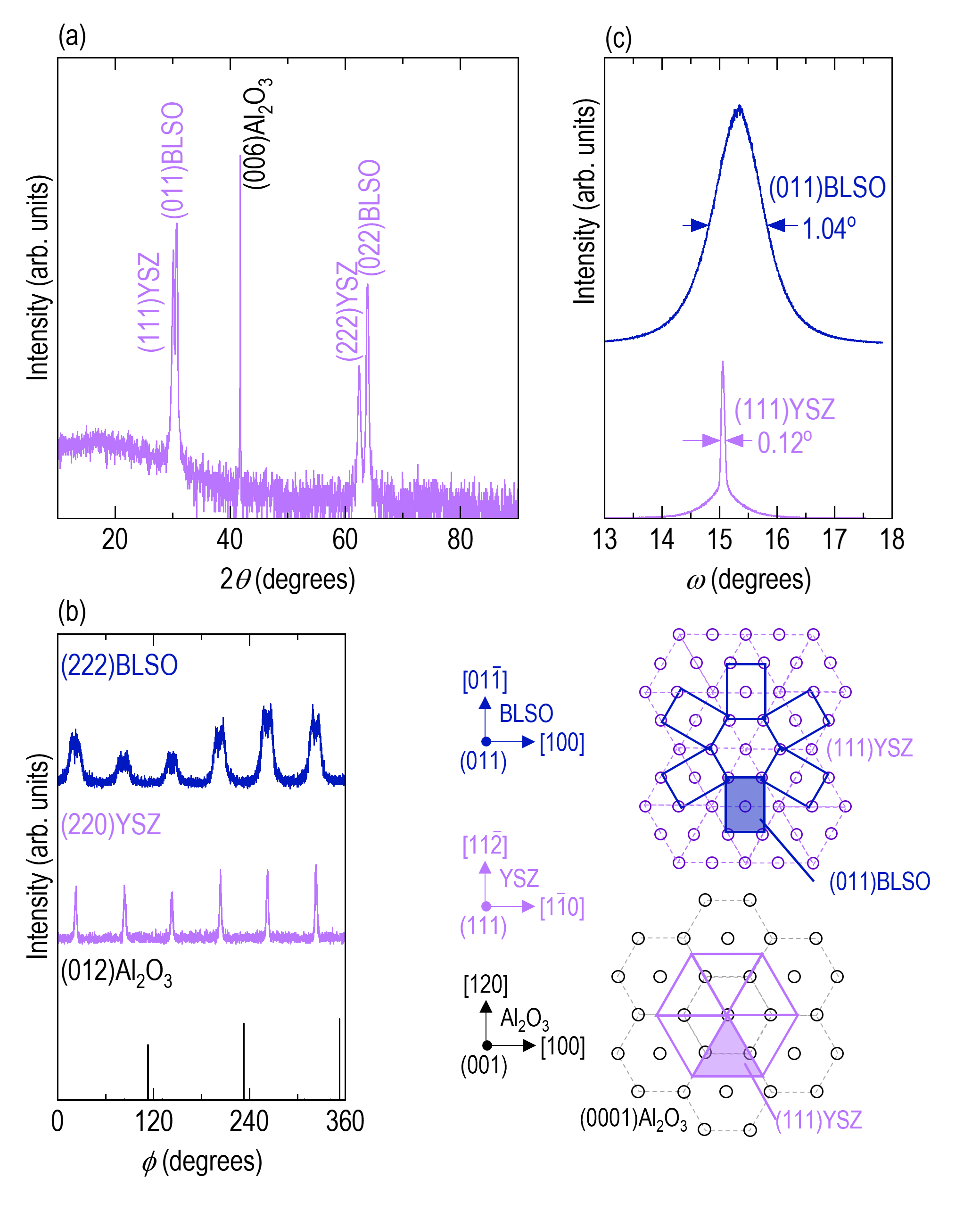
**

**Fig. S9.** (a) X-ray diffraction *θ*−2*θ* scan, (b) *φ* scan and in-plane matching, and (c) *ω* scan of $\mathrm{BLSO}_{(0001)\mathrm{Al}_{2}O_{3}}^{\mathrm{YSZ}}$.

**
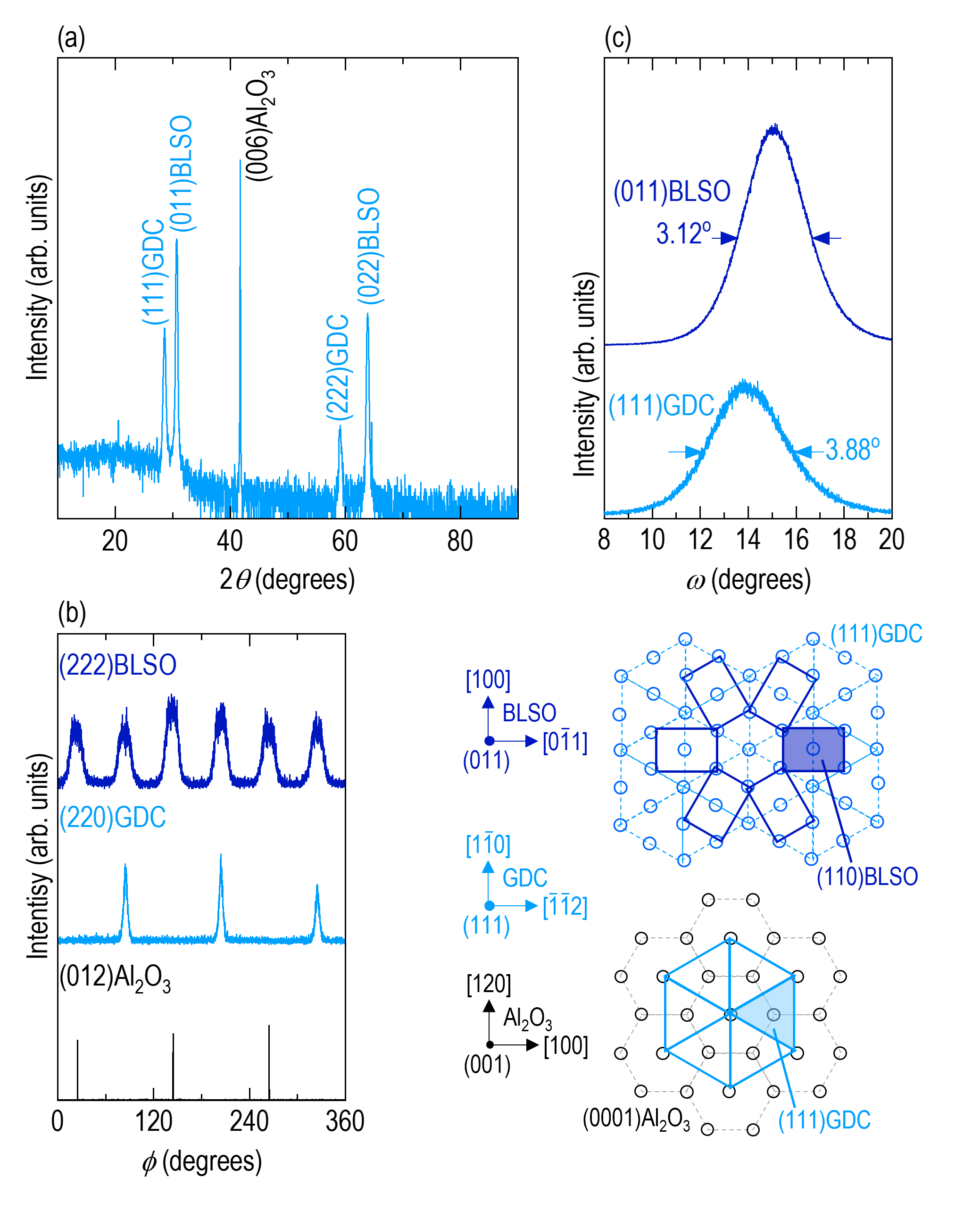
**

**Fig. S10.** (a) X-ray diffraction *θ*−2*θ* scan, (b) *φ* scan and in-plane matching, and (c) *ω* scan of $\mathrm{BLSO}_{(0001)\mathrm{Al}_{2}O_{3}}^{\mathrm{GDC}}$.

**7. Template layer thickness dependence of the electrical properties of BLSO epitaxial films**


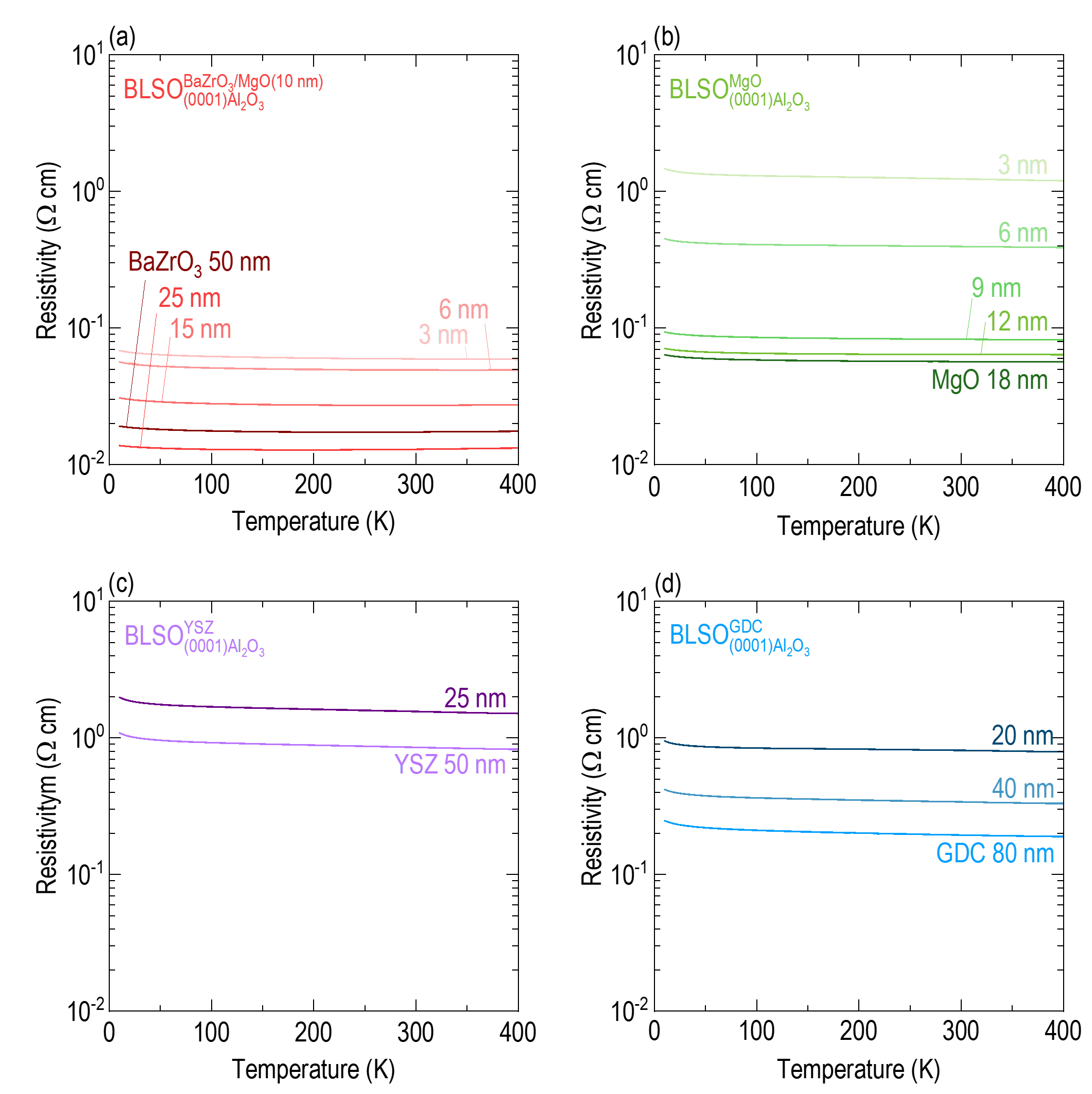


**Fig. S11.** Dependence of resistivity on BaZrO_3_ template layer thickness. (a) $\mathrm{BLSO}_{(0001)\mathrm{Al}_{2}O_{3}}^{\mathrm{BaZr}O_{3}/MgO}$, (b) $\mathrm{BLSO}_{(0001)\mathrm{Al}_{2}O_{3}}^{\mathrm{MgO}}$, (c) $\mathrm{BLSO}_{(0001)\mathrm{Al}_{2}O_{3}}^{\mathrm{YSZ}}$, and (d) $\mathrm{BLSO}_{(0001)\mathrm{Al}_{2}O_{3}}^{\mathrm{GDC}}$ The thickness of the BLSO film was 350 nm for all samples.

8. Comparison of potential materials for transparent conductors with electromagnetic shielding capabilities

**Table S2.** Resistivity (*ρ*_300 K_) and sheet resistance (*R_s_*_,300 K_) at 300 K, electromagnetic shielding effectiveness (SE_10 GHz_) at 10 GHz for the X-band, and transmittance (*T*_550 nm_) at 550 nm.

| Materials | *ρ*_300 K_  (mΩ cm) | *R_s_*_,300 K_  (Ω €^−1^) | SE_10 GHz_  (dB) | *T*_550 nm_  (%) | References |
| --- | --- | --- | --- | --- | --- |
| BLSO films grown on Al_2_O_3_ with BaZrO_3_/MgO template bilayer | 13 | 370 | 13.2 | 75 | This work |
| Carbon nanotube–polystyrene foam composites | ~1,000 | - | ~18 | - | Nano Lett. **5,** 2131 (2005) |
| Polyaniline composites  with single-wall carbon  nanotubes or graphene sheets | 50-1,000 | - | 19-29 | - | J. Phys. D: Appl. Phys. **45,** 235108 (2012) |
| Silver nanowire network | - | 15 | ~21 | - | ACS Appl. Mater. Interfaces **9,** 40857 (2017) |
| MXene forms | ~3 | - | 30-70 | - | Adv. Mater. **29,** 1702367 (2017) |
| Graphene sheets | 2-5 | - | 12-27 | - | PNAS **115,** 5359 (2018) |
| MXene-polyvinylidenefluoride | 4.7 | 2.7 | 42.9 | - | Compos. Part B-Eng. **217,** 108902 (2021) |
| MXene-CNT | 0.5 | - | 90.7 | - | J. Mater. Chem. A **9,** 24560 (2021) |
| Carbon aerogel film | 0.9 | 5.1 | 41.4 | - | Carbon **184,** 562 (2021) |

9. Total electromagnetic shielding effectiveness of $\mathbf{BLSO}_{\mathbf{(0001)}\mathbf{Al}_{\mathbf{2}}\mathbf{O}_{\mathbf{3}}}^{\mathbf{BaZr}\mathbf{O}_{\boldsymbol{3}}\mathbf{/MgO}}$


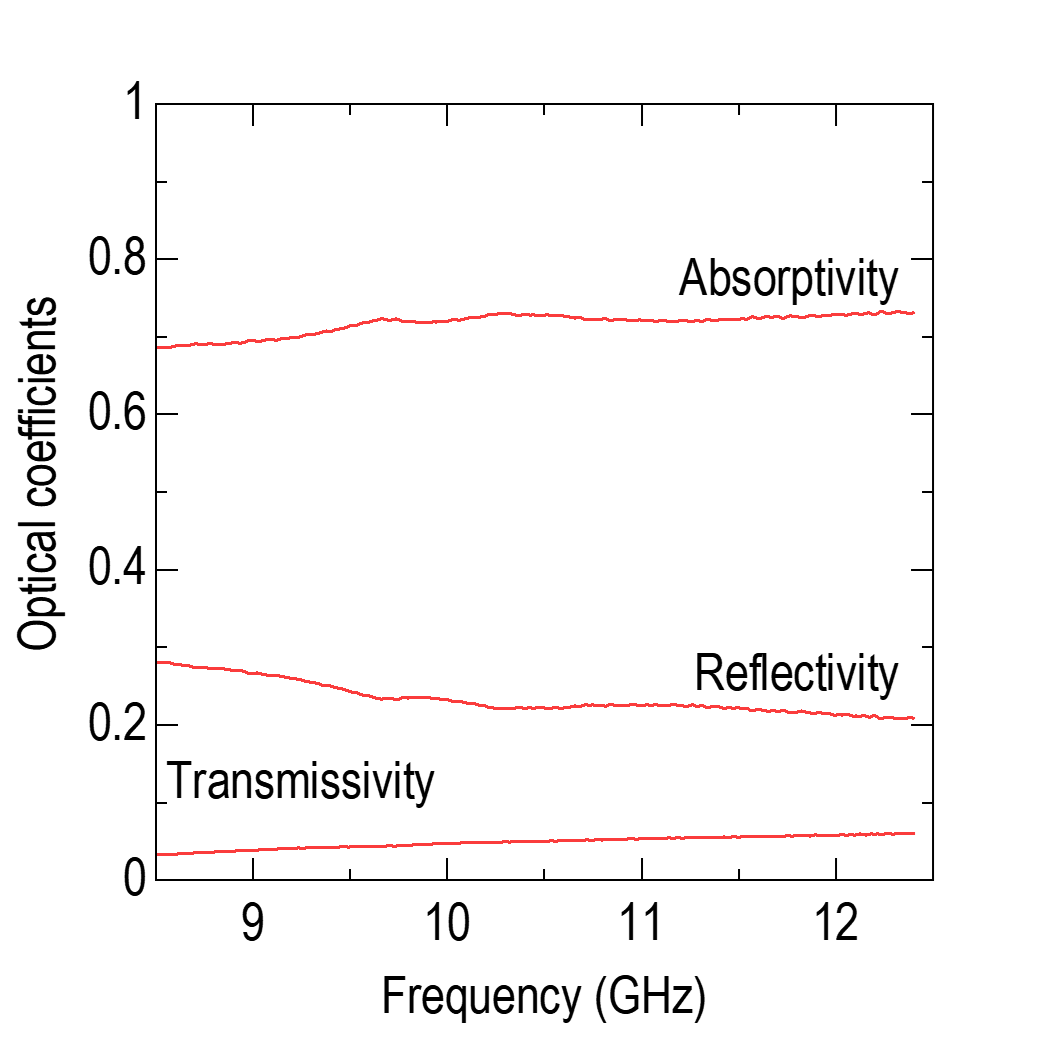


**Fig. S12**. Reflectivity and absorptivity of $\mathrm{BLSO}_{(0001)\mathrm{Al}_{2}O_{3}}^{\mathrm{BaZr}O_{3}/MgO}$. The absorptivity is much larger than the reflectivity. The transmissivity is negligible.

10. Thermal stability of $\mathbf{BLSO}_{\mathbf{(0001)}\mathbf{Al}_{\mathbf{2}}\mathbf{O}_{\mathbf{3}}}^{\mathbf{BaZr}\mathbf{O}_{\boldsymbol{3}}\mathbf{/MgO}}$ in air


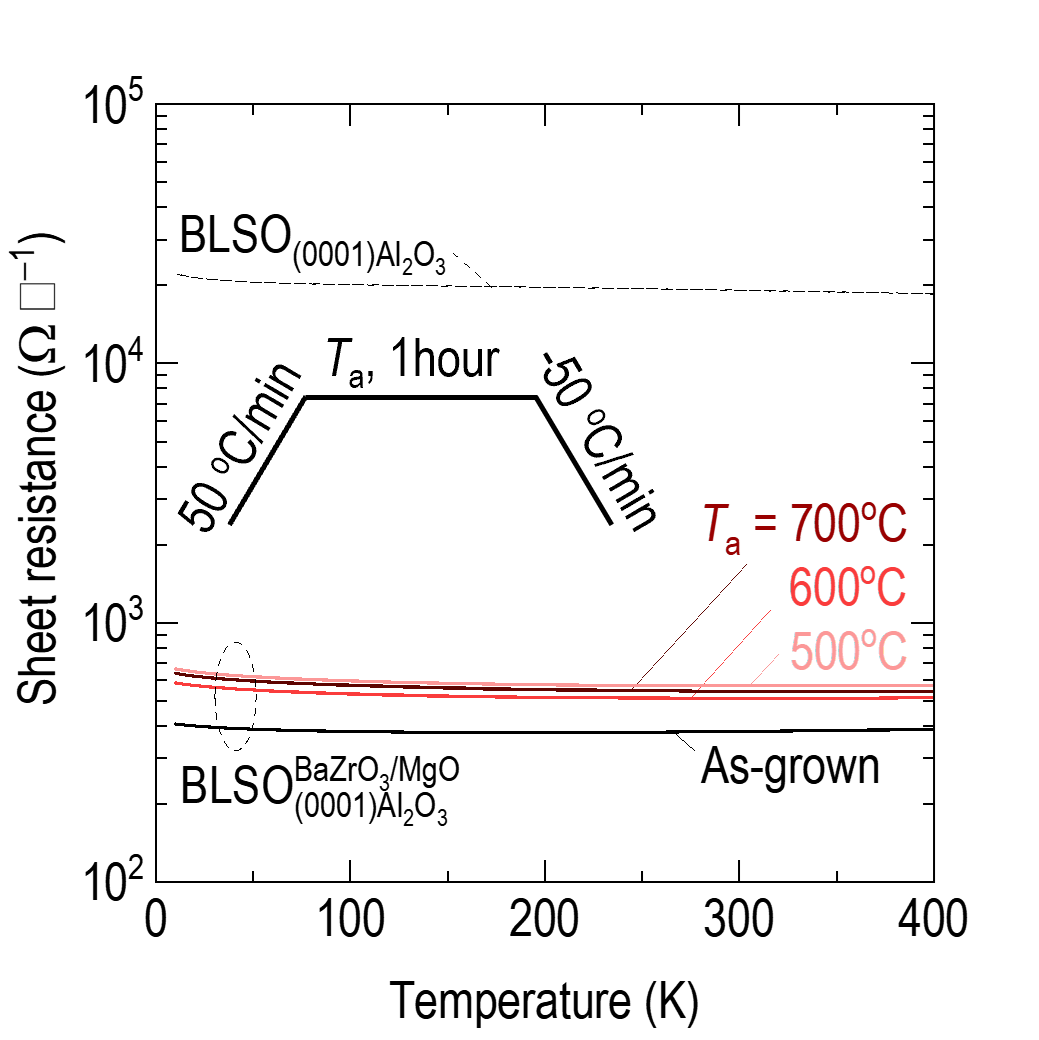


**Fig. S13**. Temperature dependence of the sheet resistance of $\mathrm{BLSO}_{(0001)\mathrm{Al}_{2}O_{3}}^{\mathrm{BaZr}O_{3}/MgO}$ after air annealing at 500, 600, and 700°C. We increased the temperature at a rate of 50°C per min, annealed the films at the annealing temperature (*T_a_*) in air for 1 h, and then decreased the temperature by 50°C per min.


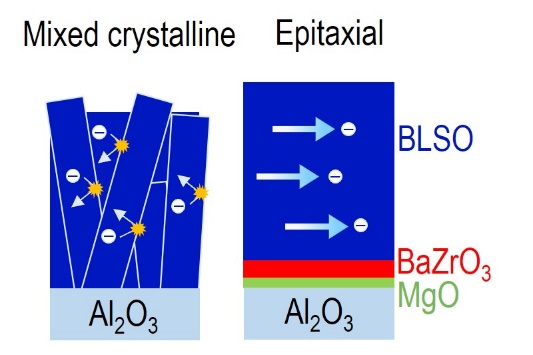

Supplement: Supplementary file 1 — Additional file 1. Additional materials, additional figures S1–S13, additional tables S1, S2. [file 40580_2023_355_MOESM1_ESM.docx]
